# Identification of Impacted Pathways and Transcriptomic Markers as Potential Mediators of Pulmonary Fibrosis in Transgenic Mice Expressing Human IGFBP5

**DOI:** 10.3390/ijms222212609

**Published:** 2021-11-22

**Authors:** Xinh-Xinh Nguyen, Ludivine Renaud, Carol Feghali-Bostwick

**Affiliations:** Division of Rheumatology & Immunology, Department of Medicine, Medical University of South Carolina, Charleston, SC 29425, USA; xinhxinhngyn@gmail.com (X.-X.N.); renaudl@musc.edu (L.R.)

**Keywords:** insulin-like growth factor protein-5 (IGFBP5), transgenic model, G-protein-coupled receptor (GPCR), neuroactive ligand receptor, fibrosis, transmembrane receptors, fibroblasts

## Abstract

Pulmonary fibrosis is a serious disease characterized by extracellular matrix (ECM) component overproduction and remodeling. Insulin-like growth factor-binding protein 5 (IGFBP5) is a conserved member of the IGFBP family of proteins that is overexpressed in fibrotic tissues and promotes fibrosis. We used RNA sequencing (RNAseq) to identify differentially expressed genes (DEGs) between primary lung fibroblasts (pFBs) of homozygous (HOMO) transgenic mice expressing human IGFBP5 (hIGFBP5) and wild type mice (WT). The results of the differential expression analysis showed 2819 DEGs in hIGFBP5 pFBs. Functional enrichment analysis confirmed the pro-fibrotic character of IGFBP5 and revealed its impact on fundamental signaling pathways, including cytokine–cytokine receptor interaction, focal adhesion, AGE-RAGE signaling, calcium signaling, and neuroactive ligand-receptor interactions, to name a few. Noticeably, 7% of the DEGs in hIGFBP5-expressing pFBs are receptors and integrins. Furthermore, hub gene analysis revealed 12 hub genes including Fpr1, Bdkrb2, Mchr1, Nmur1, Cnr2, P2ry14, and Ptger3. Validation assays were performed to complement the RNAseq data. They confirmed significant differences in the levels of the corresponding proteins in cultured pFBs. Our study provides new insights into the molecular mechanism(s) of IGFBP5-associated pulmonary fibrosis through possible receptor interactions that drive fibrosis and tissue remodeling.

## 1. Introduction

Pulmonary fibrosis (PF) is a complication of several different diseases, including systemic sclerosis (SSc aka scleroderma) and idiopathic pulmonary fibrosis (IPF). SSc is a complex autoimmune disease characterized by progressive fibrosis of the skin and multiple visceral organs due to persistent overproduction of extracellular matrix (ECM) [1,2]. Despite multiple studies, the etiology of this connective tissue disease, which causes high morbidity and mortality in the patients, is still unknown. SSc-associated pulmonary fibrosis (SSc-PF) has become the leading cause of death in SSc patients [3,4]. Similarly, IPF is associated with significant morbidity and mortality [5]. To date, FDA approved treatments only slow down the progression of PF; there is currently no effective treatment to stop or reverse the progression of fibrosis [2,3]. Therefore, identifying novel targets would significantly advance the development of effective therapeutic strategies for the treatment of PF. 

Insulin-like growth factor binding protein 5 (IGFBP5) is the most conserved member of the IGFBP family, and mouse, rat and human IGFBP5 are 97% identical [6]. IGFBP5 binds IGF1 and IGF2 with high affinity and modulates IGF signaling, either inhibiting or stimulating their effects [7]. Like other IGFBPs, IGFBP5 exerts both IGF-dependent and -independent effects [8,9]. 

Our group has reported high levels of IGFBP5 in lung tissues and primary pulmonary fibroblasts (pFBs) of SSc-PF and IPF patients as well as in dermal fibroblasts of SSc patients [10,11,12]. Additionally, we have shown that IGFBP5 induces fibrosis in vivo in mouse skin and lung [13,14] and ex vivo in human skin maintained in organ culture [9,15]. Together, these findings suggest that IGFBP5 is a key pro-fibrotic factor that is deregulated during the development and progression of SSc and IPF. 

Using CRISPR/Cas9 knock-in technology, we recently generated transgenic mice ubiquitously expressing human IGFBP5 (hIGFBP5) [16]. ECM genes, such as *Col3a1*, *Fn* and *Lox*, were highly expressed in lung and skin tissues of these mice [16]. To further determine the functional role of hIGFBP5 in the development of fibrosis, primary pFBs of wild type (WT) and homozygous (HOMO) transgenic mice were used for RNA sequencing (RNAseq). 

The differential expression (DE) analysis comparing the transcriptome of primary pFBs of hIGFBP5 HOMO mice to that of WT mice revealed 2819 differentially expressed genes (DEGs) and allowed us to conduct a full functional enrichment analysis to tease out the networks, signaling pathways and gene ontology (GO) terms impacted by hIGFBP5. To complement the RNAseq data, qRT-PCR and immunoblotting were used to confirm changes at the mRNA and protein level. Our findings provide novel insights into the role of IGFBP5 in fibrosis. 

## 2. Results

### 2.1. Transcriptomic Signature and Functional Enrichment of hIGFBP5 pFBs

**Figure 1:**
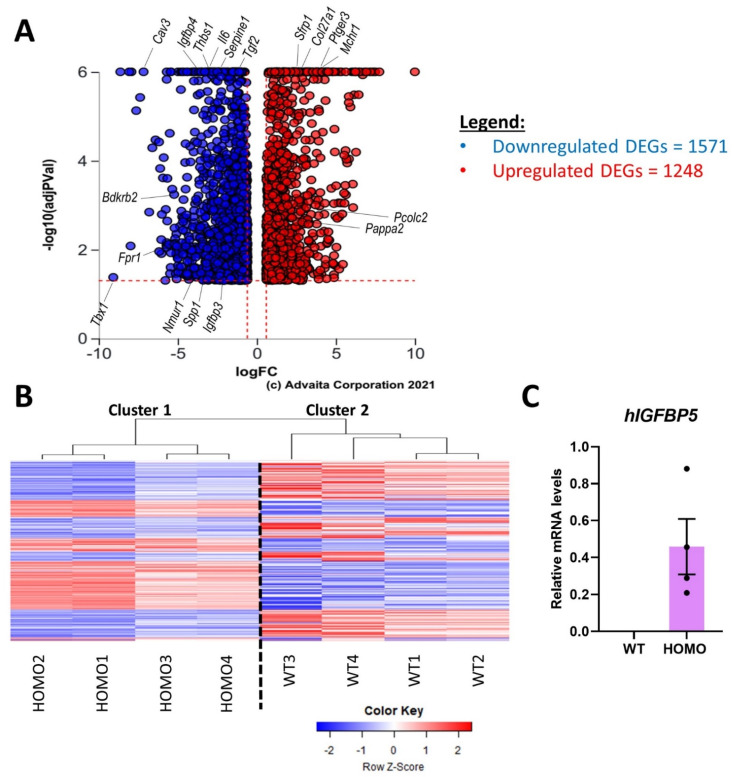
Transcriptomic signature of hIGFBP5 in pFBs. DE analysis comparing “HOMO vs. WT” mice was conducted on DESeq2 and returned 2819 DEGs. (**A**) Volcano plot showing in red DEGs significantly upregulated (*q* < 0.05; log2FC > 0.6) and in blue DEGs significantly downregulated (*q* < 0.05; log2FC < −0.6). Genes of interest are labelled. (**B**) Heat map of the DEGs in HOMO hIGFBP5 pFBs. Red: up-regulation; Blue: down-regulation. (**C**) Expression levels of transgene human *IGFBP5* (h*IGFBP5*) in the fibroblasts of female mice analyzed by RNAseq in panel B. Note that endogenous mouse *Igfbp5* was undetectable in the same samples in both WT and HOMO groups (data not shown).

The DE analysis “HOMO vs. WT” returned 2819 DEGs (1248 upregulated and 1571 downregulated genes) out of a total of 16,451 genes with measured expression (Figure 1A, Supplementary Table S1). HOMO and WT samples clustered in two separate clusters (Figure 1B), Cluster 1 and 2, respectively. As seen in Cluster 1, HOMO groups 3 and 4 had a weaker transcriptomic signature compared to HOMO groups 1 and 2. This indicated variable levels of expression of hIGFBP5 in each mouse [16]. The expression levels of the hIGFBP5 transgene were quantified in the pFBs of female mice, which were analyzed by RNAseq in panel B (Figure 1C), and as expected, levels were undetectable in pFBs of WT mice, while highly expressed in HOMO pFBs, confirming the successful CRISPR/Cas9 knock-in in the mice. Note that endogenous mouse *Igfbp5* was undetectable in the same samples in both WT and HOMO groups (data not shown). 

For the GO analysis, the “high specificity pruning” (highSP) and the “smallest common denominator pruning” (SCDP) corrections were applied to reduce redundancy and summarize the GO terms. The GO analysis (Table 1) revealed that overexpression of hIGFBP5 in fibroblasts affected “angiogenesis” and related processes (yellow), induced “positive regulation of MAPK cascade” and downstream pathways (orange), perturbed “calcium binding” (green), “ECM structural constituents” (blue) and “components of the plasma membrane”, including “receptor ligand activity” (pink). 

The pathway impact analysis uncovered 50 enriched pathways (Supplementary Table S2). Amongst the top 10 most enriched pathways, “cytokine–cytokine receptor interaction”, “focal adhesion”, “AGE-RAGE signaling pathway in diabetic complications”, “chemokine signaling pathway”, “neuroactive ligand-receptor interaction” and “calcium signaling pathway” were present (Table 1). 

In our functional enrichment analysis, the AGE-RAGE signaling pathway in diabetic complications was one of the most enriched pathways (p. 1.290E-04) (AGE: advanced glycation end-product; RAGE: receptor of AGEs). Most of the DEGs identified in this pathway were downregulated in the hIGFBP5 fibroblasts of the HOMO mice; however, the advanced glycosylation end product-specific receptor (*Ager*), the early growth response 1 (*Egr1*) transcriptional regulator, the signal transducer and activator of transcription 3 and 5a (*Stat3*, *Stat5a*), and the collagens type I alpha 1 and 2 chains (*Col1a1*, *Col1a2*) were amongst the few DEGs that were upregulated in this pathway (Supplementary Figure S1A). The coherent cascades highlight that upregulation of the *Ager* triggers upregulation of PLC (*Plce1*, *Plcb1*), which feeds into the calcium signaling pathway (p. 0.002), and upregulation of PI3K (*Pik3cb*, *Pik3r2*) as part of the PI3K-Akt signaling pathway (p. 0.003) (Supplementary Figure S1B, yellow arrows). This in turn feeds a positive feedback loop with *Ager*. Additionally, the upregulation of both *Stat3* and *Stat5a* enhance the upregulation of both collagen type 1 *Col1a1* and *Col1a2*, contributing to ECM production (Supplementary Figure S1B, yellow boxes). Finally, the upregulation of *Egr1* triggers the expression of Egr1-dependent genes and vascular dysfunction. 

The overexpression of hIGFBP5 in fibroblasts affected the expression of several structural and functional macromolecules of the ECM including the upregulation of *Col1a1*, *Col1a2*, *Col6a1*, *Col6a2*, and the downregulation of secreted phosphoprotein 1 (*Spp1*), thrombospondin 1 and 2 (*Thbs1*, *Thsb2*), laminin gamma 2 (*Lamc2*), tenascin XB and C (*Tnxb*, *Tnc*) (Supplementary Figure S2A,B). The ECM-receptor interaction pathway (p. 0.017) is disrupted in hIGFBP5 fibroblasts. This pathway leads to direct and indirect control of adhesion, migration, differentiation, proliferation, and apoptosis. Integrins are essential transmembrane receptors able to provide a force-transmitting physical link between the ECM and the cytoskeleton. We identified 5 downregulated (*Itga2/7/8*, *Itgb5/6*) and 2 upregulated (*Itga2b/3*) integrins in hIGFBP5 fibroblasts. ECM macromolecules also interact with proteoglycans and glycoproteins that are DEGs in our analysis, including the downregulated molecules *Cd36* and *Cd44*, and the upregulated glycoproteins synaptic vesicle 2a (*Sv2a*) and platelet glycoprotein 5 (*Gp5*). 

The perturbation of the ECM and the integrins we observed contributed to the deregulation of the focal adhesion pathway (Supplementary Figure S3A,B), one of the most perturbed pathways (p. 2.458E-05) in the hIGFBP5 fibroblasts. Another trigger to the enrichment of the focal adhesion pathway is the downregulation of several growth factors (GFs) and receptor tyrosine kinases (RTKs) in the cytokine–cytokine receptor interaction pathway, the most enriched pathway in our analysis (p. 6.449E-07). Lastly, the downregulation of 3 caveolin genes (*Cav1/2/3*) also contributed to the enrichment of the focal adhesion pathway. The overall perturbation of this signature predicted by coherent cascade is the upregulation of *Bcl2* associated agonist of cell death (*Bad*) (Supplementary Figure S3B, yellow arrows and box), a factor that promotes cell death in the apoptotic pathway, and the upregulation of 4 myosin light chain kinases (*Mylk1/2/3/4*), factors that play a role in the regulation of actin cytoskeleton (p. 0.029) and polymerization (Supplementary Figure S4). 

The overexpression of hIGFBP5 in fibroblasts has a profound impact on the expression of cytokines and their receptors, and the cytokine–cytokine receptor interaction pathway is the most enriched of all pathways in this functional enrichment analysis (p. 6.449E-07). Cytokines are central regulators and mobilizers of cells engaged in inflammatory host defenses (innate and adaptive), cell growth, differentiation, cell death, angiogenesis, and repair processes. In this pathway, out of 26 differentially expressed cytokines, 22 were downregulated in hIGFBP5 fibroblasts: *Ccl2/7/8/9/12/20/28*, *Il6/7/1rn*, *Tslp*, *Csf1*, *Tnfsf8/15*, *Eda*, *Ngf*, *Gdf3/6/15*, *Inhba/b* and *Tgfβ2* (Supplementary Figure S5A). The only upregulated cytokines in this pathway impacted by hIGFBP5 expression were *Cx3cl1, Gdf7*, *Il33* and *Tnfsf13*. Considering the accumulation effect of all DEGs in this pathway, the overall impact of hIGFBP5 overexpression seems to be the downregulation of many cytokine receptors, with the noticeable exception of the upregulation of receptors that interact with IL1-like cytokines, such as *Il1rap*, *Il1r1* and *Il1rl1* (Supplementary Figure S5B, yellow box). 

Perturbation of PI3K-Akt signaling is a major event in any cell, as this pathway regulates fundamental cellular functions, such as transcription, translation, proliferation, growth and survival. Here we found that hIGFBP5 expression in fibroblasts initiated the enrichment of the PI3K-Akt signaling pathway (p. 0.003) via 4 main routes (Supplementary Figure S6A,B): (1) deregulation of several GFs, including the downregulation of *Angpt2* and *Pgf*, and upregulation of the RTKs *Ntrk2* and *Erbb3*, (2) downregulation of the cytokines *Il6* and *Il7* and the cytokine receptor *Il2rg* accompanied by the upregulation of *Jak3* as part of the JAK/STAT signaling pathway, (3) the downregulation of several ECM macromolecules, including *Spp1* and *Thbs1*, and the integrins *Itga2/7/8* and *Itgb5/6* as part of the focal adhesion pathway (Supplementary Figures S3 and S4) upregulation of the GPCR *Lpar2* as part of the chemokine signaling pathway (p. 7.482E-04) (Supplementary Figure S7). The resulting accumulation on the PI3K-Akt pathway as predicted by iPathwayGuide would affect several pathways and GO processes that we identified as significantly enriched, namely cell proliferation (p. 4.07E-02) and angiogenesis (p. 3.67E-04) via downregulation of *Map2k1/2* (MEK) and *Mapk1/3* (ERK) as part of the MAPK signaling pathway (p. 0.013) (Supplementary Figure S8), as well as cell survival via upregulation of *Bcl2l1* and *Myb*. 

As previously mentioned, the calcium signaling pathway (p. 0.002) is among the top 10 most enriched pathways in hIGFBP5 fibroblasts (Supplementary Figure S9A,B). The downregulation of several receptor-operated calcium channels (ROCs) initiates this perturbation, such as glutamate ionotropic receptor NMDA type subunit 2a (*Grin2a*) and purinergic receptor P2X 7 (*P2rx7*). The deregulation of several voltage-dependent calcium channels also contributes to the enrichment of this pathway, including the upregulation of the L type, alpha 1c and 1i subunits (*Cacna1c/1i*) and the downregulation of the P/Q type, alpha 1A subunit (*Cacna1a*), as well as the downregulation of the ORAI calcium release-activated calcium modulators 1 and 3 (*Orai1/3*). The downregulation of several *GPCRs* also contributes to this perturbation, including angiotensin II receptor type 1b (*Agtr1b*), bradykinin receptor beta 2 (*Bdkrb2*), chemokine C-X-C motif receptor 4 (*Cxcr4*), prostaglandin F receptor (*Ptgfr*), histamine receptor H1 (*Hrh1*), 5-hydroxytryptamine (serotonin) receptor 2A (*Htr2a*), adrenergic receptors alpha 1b/1d and beta 2 (*Adra1b/1d*, *Adrb2*), adenosine A2b receptor (*Adora2b*). Note that the prostaglandin E receptor 3 (*Ptger3*) is the only GPCR that is upregulated in this pathway. Two RTKs are also involved, the upregulated erb-b2 receptor tyrosine kinase 3 (*Erbb3*) and the downregulated platelet derived growth factor receptor beta (*Pdgfrb*). *CD38*, a transmembrane glycoprotein, is defined as both a cell surface enzyme and as a receptor that also participates in the calcium signaling enrichment (see ADPR cyclase) because it is upregulated in hIGFBP5 fibroblasts. Noticeably, ligands to the GPCRs and the ROCs include neurotransmitters. 

Interestingly, the **neuroactive ligand-receptor interaction** pathway is also amongst the top 10 most enriched pathways (p. 0.001) and many GPCRs that respond to different types of ligands are involved in this enrichment (Supplementary Figure S10A,B). Downregulated GPCRs in this pathway are often those responsive to Class A rhodopsin-like amines (*Adra1b/1d*, *Adrb2*, *Hrh1*, *Htr2a*), peptides (*Agtr1b*, *Bdkrb2*, *C3ar1*, *C5ar1*, *Fpr1*, *Nmur1*), hormone proteins (*Tshr*), prostanoids (*Ptgfr*), nucleotide-like ligands (*Adora2b*, *P2ry2/6/14*), cannabinoids (*Cnr2*), lysophosphatidic acid and LPA (*S1pr1*) and class B secretin-like ligands (*Adcyap1r1* labelled PACAPR). Nevertheless, few GPCRs are upregulated, including *Kissr1*, *Mchr1*, *Ptger3*, *Lpar2*, and *Gabbr1*. Additionally, several channels and other types of receptors are also perturbed in hIGFBP5 fibroblasts, including the downregulation of *Grin2a/3a*, *Gria4*, *P2rx7* and *Tspo*, and the upregulation of *Chrna4* and *Thrb*. 

One particular aspect of the impact of hIGFBP5 overexpression on the transcriptome of pFBs was the deregulation of many receptors and integrins. Out of 2819 DEGs, 199 were receptors (including 7 integrins) representing 7.1% of the transcriptome of the hIGFBP5 pFBs (Supplementary Table S3). Out of these 199 receptors, 124 were downregulated and 75 were upregulated by hIGFBP5 overexpression. This impact on receptors is reflected in the enrichment of GO terms and pathways, such as receptor ligand activity, integral component of plasma membrane, cytokine–cytokine receptor interaction, viral protein interaction with cytokine and cytokine receptor, and neuroactive ligand-receptor interaction, to name a few (Table 1). By entering the list of differentially expressed receptors from Supplementary Table S3 into ToppFun (ToppGene Suite), the output reinforced that G protein-coupled receptor, immune and cytokine receptor, steroid hormone receptor activities and ECM binding are highly perturbed in hIGFBP5 pFBs (Supplementary Table S4). 

Characterization of the transcriptomic signature and the functional enrichment of overexpression of hIGFBP5 in murine pFBs provides insights into the effects of IGFBP5 and complements our previous studies on the functional impact of IGFBP5 [9,11,12,13,14,15,16,17,18]. Here, we confirmed that IGFBP5 is pro-fibrotic, and we also uncovered novel properties by analyzing its effect at the systems level. 

### 2.2. Hub Gene Analysis

**Figure 2:**
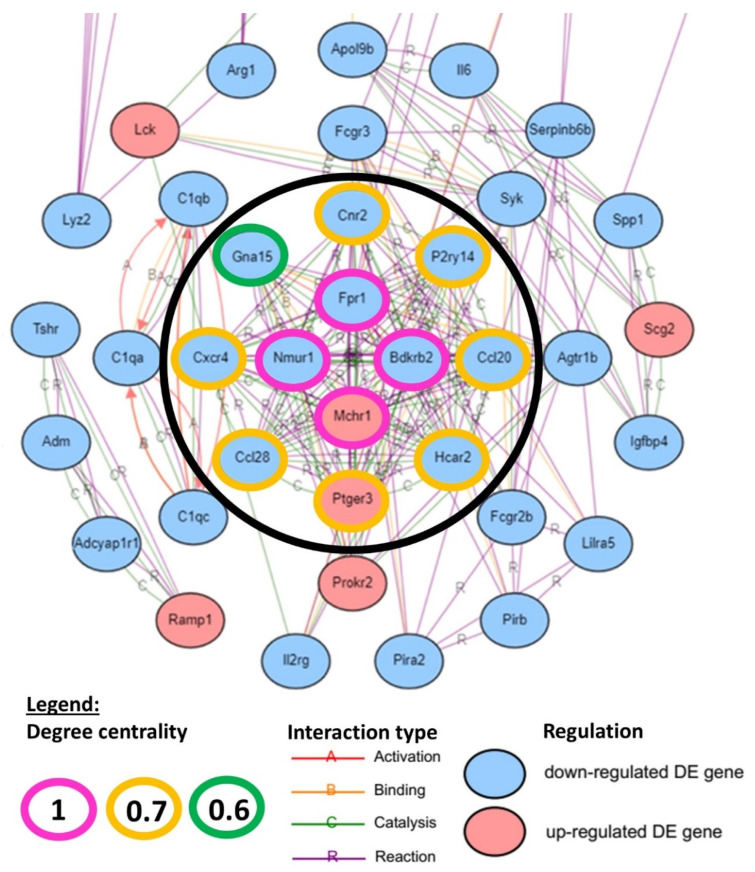
Hub gene network. DEGs with the highest degree of centrality are shown in the center of the wheel-shaped network within the black circle. Pink indicates DEGs with a degree equal to 1. Orange indicates DEGs with a degree equal to 0.7, and green indicates DEGs with a degree equal to 0.6. Genes that are upregulated in hIGFBP5 fibroblasts are shown in red, those that are downregulated are shown in blue.

A hub gene network was constructed with iPathwayGuide to evaluate the centrality degree of each DEG in hIGFBP5-expressing fibroblasts. The input genes included 500 genes with the highest absolute value of log fold change from all DE genes (Supplementary Table S5). The hub gene analysis revealed 12 hub genes with a centrality degree ≥ 0.6 (Figure 2). The hub genes with the highest centrality degree of “1” were the formyl peptide receptor 1 (*Fpr1*), the bradykinin receptor beta 2 (*Bdkrb2*), the melanin-concentrating hormone receptor 1 (*Mchr1*) and the neuromedin U receptor 1 (*Nmur1*) (pink outlines). Other hub genes with a centrality degree between “0.6 and 0.7” were the cannabinoid receptor 2 (*Cnr2*), the purinergic receptor P2Y14 (*P2ry14*), the C-C motif chemokine ligands 20 (*Ccl20*) and 28 (*Ccl28*), the hydroxycarboxylic acid receptor 2 (*Hcar2*), the prostaglandin E receptor 3 (*Ptger3*), the C-X-C motif chemokine receptor 4 (*Cxcr4*) and the G protein subunit alpha 15 (*Gna15*) (orange–green outlines). Note that all these hub genes are downregulated in hIGFBP5 pFBs, except for *Mchr1* and *Ptger3* which are upregulated (Figure 2). 

Next, we aimed to determine in which enriched pathways the identified hub genes belonged (Table 2). *Cxcr4*, *Ccl20* and *Ccl28* are present in the cytokine–cytokine receptor interaction (Supplementary Figure S5B,C) and chemokine signaling pathways (Supplementary Figure S7B,C). *Fpr1*, *Bdkrb2*, *Mchr1*, *Nmur1*, *Cnr2*, *P2ry14* and *Ptger3* all belong in the neuroactive ligand-receptor interaction pathway (Supplementary Figure S10B,C), and *Gna15*, *Bdkrb2*, *Ptger3* and *Cxcr4* in the calcium signaling pathway (Supplementary Figure S9B,C). The hub gene *Cxcr4* belongs to the axon guidance pathway; *Bdkrb2* belongs to complement and coagulation cascades, the cGMP-PKG pathway, the regulation of actin cytoskeleton (Supplementary Figure S4B,C) and endocrine and other factor-regulated calcium reabsorption pathways. *Fpr1* is part of the Rap1 pathway, and *Ccl20* of the TNF pathway. The GPCR hub genes *Hcar2* and *Ptger3* also belong to the cAMP signaling pathway, a pathway that is not significantly enriched in our analysis (p. 0.487). 

Taken together, the findings of the hub gene analysis emphasize that expression of hIGFBP5 in fibroblasts affects the expression of several hub genes that are involved in pathways that have far reaching outcomes in the regulation of fundamental processes, and that the hub gene *Bdkrb2* is involved in six enriched pathways, highlighting its importance in hIGFBP5 pFBs. 

### 2.3. Sex Affects the Impact of hIGFBP5 on the mRNA Levels of Genes of Interest in pFBs

**Figure 3:**
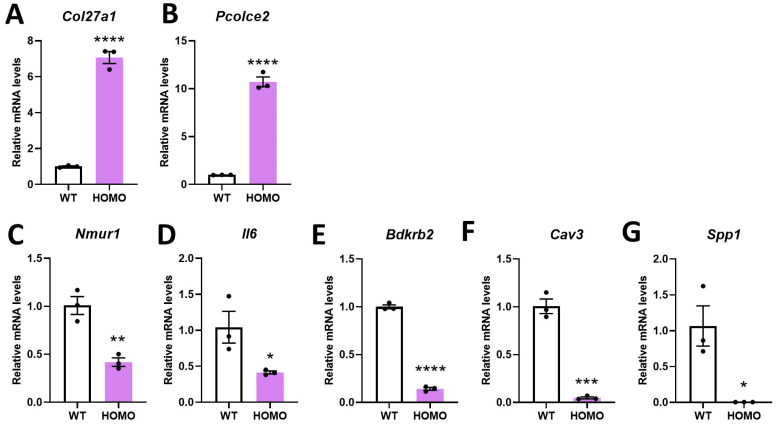
Impact of hIGFBP5 on the mRNA levels of genes of interest in pFBs. Expression levels of genes of interest were quantified by qRT-PCR. (**A**) *Col27a1*. (**B**) *Pcolc2*. (**C**) *Nmur1*. (**D**) *Il6*. (**E**) *Bdkrb2*. (**F**) *Cav3*. (**G**) *Spp1*. n = 3 per group. The housekeeping gene used to normalize the data was *B2m*. * *p* < 0.05. ** *p* < 0.01. *** *p* < 0.001. **** *p* < 0.0001. WT: wild type female mice, HOMO: hIGFBP5 homozygous female mice.

To complement the RNAseq data obtained in HOMO and WT female mice, several gene of interests (GOIs) were analyzed by qRT-PCR using fibroblasts from both male and female mice. The results in female mice are presented in Figure 3, and the results for both sexes normalized to female WT mice are presented in Supplementary Figure S11. 

In hIGFBP5-expressing fibroblasts of HOMO female mice, the expression levels of *Col27a1* and *Pcolce2* were increased compared to WT mice (Figure 3A,B), while *Nmur1*, *Il6*, *Bdkrb2*, *Cav3* and *Spp1* were significantly decreased (Figure 3C–G)-regulation that is consistent with our RNAseq data (Table 3). In the fibroblasts of HOMO male mice, expression of hIGFBP5 had no significant effect on the expression of *Col27a1*, *Pcolce2*, *Bdkrb2*, *Cav3* and *Spp1* (Supplementary Figure S11A–E, green bars). Only *Nmur1* and *Il6* were significantly downregulated by the expression of hIGFBP5 in fibroblasts from both female and male HOMO mice (Supplementary Figure S11F,G). Note that the expression levels of all these GOIs were significantly different in female and male WT mice, while *Col27a1*, *Pcolce2*, *Cav3* and *Il6* expression was different in fibroblasts from HOMO female and male mice (Supplementary Figure S11A,B,D,G). Overall, the results of the RNAseq and qRT-PCR complement each other and highlight the importance that sex has on the effect of hIGFBP5 expression in pFBs. 

### 2.4. Effect of hIGFBP5 on Intracellular Protein Abundance

**Figure 4:**
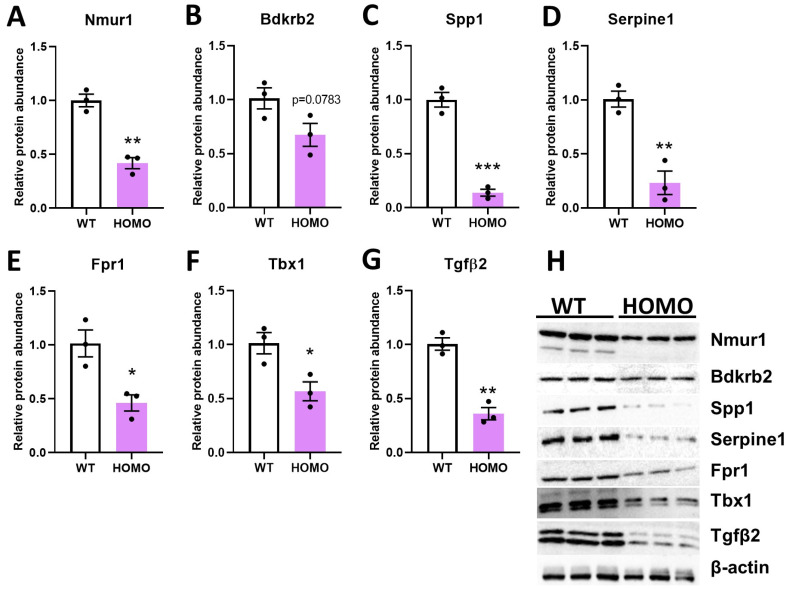
Effect of hIGFBP5 on Intracellular Protein Abundance. Cell lysates were analyzed by immunoblotting for selected proteins. Quantification of immunoblots (n = 3) for the protein abundance of (**A**) Nmur1, (**B**) Bdkrb2, (**C**) Spp1, (**D**) Serpine1, (**E**) Fpr1, (**F**) Tbx1 and (**G**) Tgfβ2. The housekeeping protein used to normalize the data was β-actin. * *p* < 0.05. ** *p* < 0.01. *** *p* < 0.001. WT: wild type female mice, HOMO: hIGFBP5 homozygous female mice. (**H**) Representative immunoblots for each protein shown in panels A-H and β-actin as a loading control.

In cell lysates from hIGFBP5 pFBs of HOMO female mice, the abundance of selected proteins was quantified by immunoblotting. We determined that expressing hIGFBP5 considerably decreased the intracellular protein abundance of Nmur1, Bdkrb2, Spp1, Serpine1, Fpr1, Tbx1 and Tgfβ2 in pFBs of HOMO female mice (Figure 4A–H). Our observations at the protein level in cell lysates were consistent with the RNAseq data (Table 3). In contrast, hIGFBP5 expression had no effect on Nmur1, Bdkrb2, Serpine1, Fpr1 and Tbx1 protein abundance in the lysates of HOMO male pFBs (Supplementary Figure S12A–F, green bars). It is worth noting that Tbx1 levels were significantly different between female and male WT pFBs (Supplementary Figure S12E,F). 

### 2.5. Effect of hIGFBP5 on the Secretion of Igfbp4, Serpine1 and Pappa2

**Figure 5:**
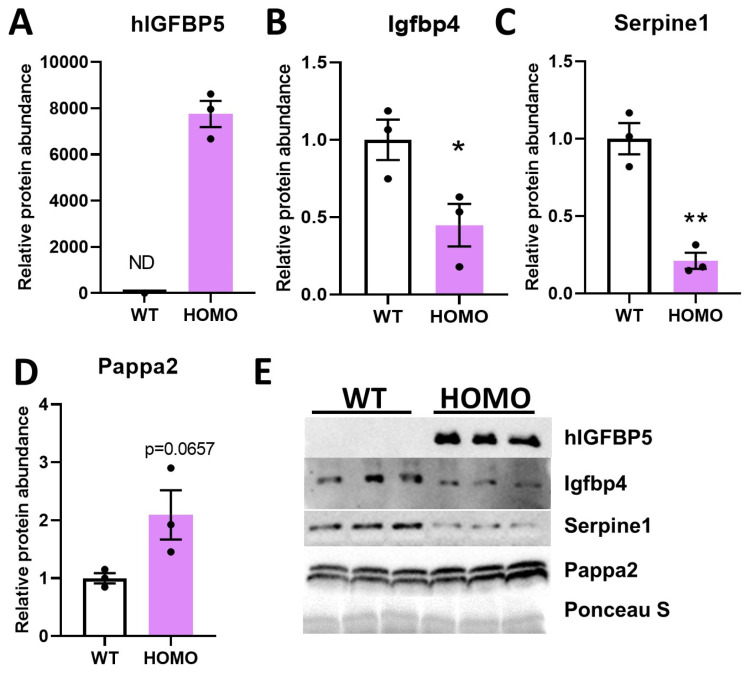
Effect of hIGFBP5 on the secretion of Igfbp4, Serpine1 and Pappa2. Conditioned media of cultured pFBs of hIGFBP5 HOMO and WT mice were analyzed by immunoblotting for selected proteins. Quantification of immunoblots (n = 3) for the protein abundance of (**A**) hIGFBP5, (**B**) Igfbp4, (**C**) Serpine1 and (**D**) Pappa2. * *p* < 0.05. ** *p* < 0.01. ND: non-detectable. WT: wild type female mice, HOMO: hIGFBP5 homozygous female mice. (**E**) Representative immunoblots showing protein abundance of hIGFBP5, Igfbp4, Serpine1 and Pappa2 in conditioned media of cultured pFBs of hIGFBP5 HOMO and WT mice. Ponceau S staining was used as loading control.

Our RNAseq data showed that expression of hIGFBP5 in pFBs downregulated the expression levels of other IGFBPs such as *Igfbp4* and *Igfbp3* (Table 3). Secreted hIGFBP5 was detected at high levels in the conditioned media of hIGFBP5 pFBs, and, as expected, was not detectable in the media of WT fibroblasts (Figure 5A,E). Protein abundance of Igfbp4 and Serpine1 in conditioned media were significantly decreased in hIGFBP5 pFBs of female mice. However, secretion of pappalysin 2 (Pappa2), a metalloproteinase that specifically cleaves Igfbp5 [19], was markedly increased in hIGFBP5 pFBs (Figure 5D,E). These results are consistent with the RNAseq data (Table 3). These changes were also noted in the pFBs of male mice, but the difference did not reach statistical significance (Figure S13). In addition, Igfbp4 and Serpine1 levels were significantly different between female and male WT pFBs (Supplementary Figure S13A,B,D). 

## 3. Discussion

In the current study, we present a global functional enrichment of the impact of the expression of hIGFBP5 on the transcriptome of primary pFBs of HOMO mice. We have previously shown that adenoviral overexpression of IGFBP5 or recombinant IGFBP5 induced a fibrotic phenotype in pFBs by increasing the expression levels of *COL1a1*, *CTGF*, *LOX*, *FN*, *Acta2*, *EGR1* and *DOK5* in a MAPK- and EGR1-dependent manner [11,17,18]. We have also shown that IGFBP5 promotes fibrosis by increasing its own expression [11]. To achieve a more physiologically relevant model and study the exogenous effects of IGFBP5, we generated transgenic mice ubiquitously expressing hIGFBP5 using CRISPR/Cas9 knock-in [16]. 

### 3.1. Effect of hIGFBP5 on ECM Macromolecules and ECM-Receptor Interaction

The expression levels of several collagens and ECM macromolecules were affected by the expression of hIGFBP5 in pFBs leading to the enrichment of the ECM-receptor pathway. The fibrillar collagens *Col1a1* and *Col1a2* were upregulated, in agreement with our previous findings [16], and we also identified the upregulation of non-fibrillar *Col6a1* and *Col6a2*. Several other collagens were affected by the overexpression of hIGFBP5 in pFBs; some were upregulated, including *Col11a2*, *Col20a1* and *Col27a1*, and some were downregulated, such as *Col5a1*, *Col8a1*, *Col12a1*, *Col14a1*, *Col18a1* and *Col19a1*. The function of these collagens has not been fully characterized in PF yet, however increased levels of COL11A2 and COL27A1 have been reported in cardiac fibrosis and IPF, respectively [20,21]. Paraquat, which causes PF, was shown recently to upregulate the newly described fibrillar collagen COL27A1 in human pFBs [22,23]. This is in line with the pro-fibrotic character of hIGFBP5, which induced these collagens in pFBs. However, hIGFBP5 also downregulated *Col5a1*, *Col8a1*, *Col12a1*, *Col14a1*, *Col18a1* and *Col19a1*, and to better understand that outcome, a characterization of the levels and roles of each of these collagens in PF is needed. 

Other ECM macromolecules were downregulated by the expression of hIGFBP5 in fibroblasts, such as *Ctgf*, *Spp1*, *Lamc*, *Thbs1*, *Thsb2*, *Tnxb* and *Tnc*. Thbs1 is a double facetted glycoprotein that can either stimulate or inhibit angiogenesis and wound healing [24]. Thbs1 null mice have impaired lung homeostasis that render them more prone to lung injury due to defective Il-10 signaling, emphasizing the role Thbs1 plays in providing an appropriate inflammatory response [25]. Increased levels of CTGF, SPP1, THBS2, TNXB, TNC are associated with PF mostly in response to TGFβ signaling [26,27,28,29,30]. Since hIGFBP5 did not affect *Tgfβ1* levels, and downregulated *Tgfβ2* levels, the downregulation of these ECM macromolecules that are downstream targets of TGFβ1 and 2 is sensible. In fact, some have wondered if TGFβ and IGFBP5 are redundant or complementary to each other in their mode of action [31]. 

Interestingly, IGFBP5 shares structural similarities with CTGF, and, historically, secreted cysteine-rich (CCN) proteins were named IGFBP8-10 [31]. A regulatory mechanism that downregulates *Ctgf* when hIGFBP5 is overexpressed may be in place to prevent redundancy or reflect a negative feedback loop. Validation experiments are necessary to fully comprehend this transcriptomic signature and regulatory mechanisms in hIGFBP5 pFBs. 

Overexpression of hIGFBP5 in FBs upregulated *Pcolce2*, a collagen-binding protein that can bind to heparin with great affinity [32]. In Pcolce2 null mice undergoing pressure-overload induced cardiac remodeling, collagen accumulation was significantly reduced compared to WT mice, emphasizing the importance of Pcolce2 in procollagen post-synthetic processing in the regulation of collagen accumulation in the heart [33]. This study highlighted Pcolce2 as a potential marker to monitor collagen accumulation or to serve as a target to attenuate cardiac fibrosis [33]. 

Taken together, our data showed that hIGFBP5 induced the expression levels of several known collagens and ECM macromolecules as well as yet uncharacterized ones, confirming our previous studies on the role of IGFBP5 in the development of fibrosis [11,12,16]. 

### 3.2. Effect of hIGFBP5 on AGE-RAGE and Egr1-MAPK Dependent Signaling Pathways

Perturbation of the AGE-RAGE signaling pathway has been well characterized in the pathogenesis of diabetes [34], a signature that was highlighted in our functional enrichment analysis of hIGFBP5 fibroblasts. More recently, the AGE-RAGE signaling pathway has been implicated in aging diseases and IPF [35,36]. In fact AGEs have been described as biomarkers of aging processes that contribute to pulmonary fibrosis [37]. 

Although most of the DEGs we identified in this pathway were downregulated in hIGFBP5 fibroblasts, *Ager*, *Egr1*, *Stat3*, *Stat5a*, *Col1a1* and *Col1a2* were upregulated. The link between IGFBP5 and Ager has not been reported in the literature. However, we have previously shown that IGFBP5 induced a fibrotic phenotype in lung tissues and pFBs of IPF patients by upregulating the transcription factor EGR1, a key player in the development of fibrosis, in a MAPK-dependent manner [17]. These findings were also observed in hIGFBP5 pFBs as the MAPK signaling pathway was significantly enriched and Egr1 expression was upregulated. In the hIGFBP5 pFBs, *Stat3* was upregulated, a signature that leads to lung myofibroblast differentiation and the production of collagen type 1 and ECM [38,39]. 

Through the enrichment of ECM macromolecules, AGE-RAGE and Egr1-MAPK dependent signaling pathways, hIGFBP5 hijacked fundamental pathways and biological processes such as PI3K-Akt, calcium, focal adhesions pathways and vascular dysfunction GO term. In fact, angiogenesis, regulation of blood coagulation and morphogenesis of a branching structure were enriched biological process terms (Table 1). 

### 3.3. Effect of hIGFBP5 on Focal Adhesion, Calcium Signaling and Actin Polymerization

The enrichment of both the ECM-receptor and cytokine–cytokine receptor interaction pathways led to perturbation of focal adhesion in hIGFBP5 pFBs, a pathway that is involved in the development of PF [40]. The observed transcriptomic signature of hIGFBP5 pFBs is predicted by the perturbation cascade resulting in upregulation of *Mylk1-4* and *Bad*, affecting the regulation of actin cytoskeleton and cell survival. 

Nonmuscle myosin II filaments, along with actin filaments and α-actinin, are contractile stress fibers prominent in fibroblasts [41]. Myosin light chain kinases (Mylks aka MLCK) are Ca^2+^-calmodulin (CaM)-dependent enzymes that phosphorylate and activate myosin II and are involved in the formation and disassembly of focal adhesions [42]. Inhibition of CaM-MLCK has been shown to affect fibroblast-populated collagen lattice contraction, cell migration, focal adhesion and wound contraction [43]. This also emphasizes the importance of intracellular calcium in the regulation of cellular mechanics as calcium and CaM activate MLCK, which in turn activates myosin II in the contracting tails during locomotion [44]. The calcium signaling pathway was enriched in our analysis. It has been reported that calcium oscillations play a central role in the regulation of gene expression in human pFBs [45] and that disruption of the calcium signaling in FBs protected against PF [46]. 

The actin cytoskeleton of FBs provides information about the physical environment in which they are, information that is crucial for contractility and motility, and cytoskeletal stiffness is increased in PF, possibly contributing to lung stiffness [47]. Regulation of the actin cytoskeleton pathway was enriched in our analysis, and the downregulation of the hub gene *Bdkrb2* is predicted to have an impact by contributing to the downregulation of *Rac1/2/3*, leading to the upregulation of the *Mylk1-4* genes discussed above, and the upregulation of *Spata13* and *Arhgef4* (labelled Asef), *Cyfip1/2* (labelled PIR121), *Nckap1* (labelled Nap125) and *Abi2*, affecting actin polymerization. In SSc dermal FBs, the actin cytoskeleton pathway was also enriched [48], and IGFBP5 has been shown to rapidly reorganize the cytoskeleton of mesangial cells and stimulate their migration [49]. Taken together, our results suggest that hIGFBP5 is involved in the regulation of focal adhesion and actin polymerization in a calcium-dependent manner in response to changes in the ECM, an effect that impacts cell migration. 

### 3.4. Effect of hIGFBP5 on PI3K-Akt, Cell Survival, and Angiogenesis

Cell survival and apoptosis are major outcomes of the PI3K-Akt pathway, as well as the perturbation cascade of the focal adhesion pathway downstream of bad upregulation. The IGFBP5-STAT3 axis has been shown to induce cell survival and proliferation in human FBs [50] and in hIGBFP5 pFBs, *Stat3* was upregulated. Surprisingly, *Il6* was downregulated in our dataset, in contrast to findings by Kojima et al. [50]. On the other hand, *Il33* was significantly upregulated in hIGFBP5 pFBs. IL33 is a nuclear cytokine that is released by necrotic cells in damaged tissues to modulate inflammation and survival in FBs [51,52]. Additionally, IL33 is implicated in the development of PF [53]. Our findings suggest that IGFBP5 is upstream of IL33, which may contribute to its pro-fibrotic properties. 

Another perturbation outcome on the PI3K-Akt pathway is on cell proliferation, angiogenesis, and DNA repair via the downregulation of the Grb2-SOS-Ras-Raf1-MEK-ERK axis. The role of angiogenesis in PF has generated some debate: some argue that angiogenesis is increased in the fibrotic lung while others have reported a decrease in the most fibrotic regions of the lung [48,54]. In cancer, IGFBP5 is characterized as an inhibitor of angiogenesis, contributing to tumor suppression [54]. Since the role of angiogenesis in PF is unresolved, it is hard to conclude how the signature captured in hIGFBP5 pFBs may contribute to PF. Fully characterizing the role of angiogenesis in PF is necessary to better understand the role that IGFBP5 plays in this context. Nevertheless, our data suggest that the transcriptomic signature of hIGFBP5 pFBs may support the view that IGFBP5 is both an anti-angiogenic and pro-fibrotic factor. 

### 3.5. hIGFBP5 Downregulated Igfbp3, Igfbp4 and Igfbp7

The overexpression of hIGFBP5 in pFBs decreased the mRNA levels of three other IGFBPs: *Igfbp3*, *Igfbp4* and *Igfbp7*. Note, however, that endogenous mouse *Igfbp5* mRNA levels were not affected by hIGFBP5. In conditioned media, the abundance of hIGFBP5 was greatly increased and that of Igfbp4 was decreased while the levels of Igfbp3 were unaltered. For *Igfbp3* and *Igfbp7*, this signature is surprising as we have previously reported that these genes are upregulated in SSc-PF and IPF pFBs [55]. However the decrease of *Igfbp4* levels is consistent with our previous reports demonstrating IGFBP4 was downregulated in SSc-PF pFBs [56] and its reduction may serve to prevent its anti-fibrotic effects. 

Kojima et al. reported that IL6 stimulation increased IGFBP5 levels but did not increase those of IGFBP3 or IGFBP7 [50]. Clearly different regulatory mechanisms are in place for each IGFBP, and the downregulation of *Il6* in hIGFBP5 pFBs might explain why murine *Igfbp5* was not induced. 

We have previously shown that TGFβ1 stimulation in pFBs increased levels of IGFBP3 without affecting those of IGFBP5 [12]. In hIGFBP5 pFBs, *Tgfβ1* was not differentially expressed and *Tgfβ2* was downregulated, prompting us to speculate that hIGFBP5 may downregulate *Igfbp3* by decreasing *Tgfβ2*. Nevertheless, the observation that *Igfbp3* is downregulated when hIGFBP5 is highly expressed is in line with previous studies [12,50]. 

### 3.6. Effect of hIGFBP5 on Neuroactive Ligand-Receptor Interaction

The neuroactive ligand-receptor interaction pathway was part of the top 10 most enriched pathways due to the downregulation of several GPCRs involved in this pathway, and the upregulation of a handful of others including *Kissr1*, *Mchr1*, *Ptger3*, *Lpar2*, and *Gabbr1*. In IPF tissues, this pathway is also enriched, mainly due to the downregulation of several of its DEGs [57]. However, the upregulation of *MCHR1* was also reported in pFBs of IPF patients [58] and most recently in our data using dermal fibroblasts from twins discordant for SSc [59], consistent with our current findings. 

The lungs contain sensory nerves that play an important role in regulating cardiopulmonary functions and maintaining homeostasis in healthy and diseased states [60]. The fact that hIGFBP5 affects the expression of many receptors and their ligands in this pathway may be attributed to communication between pFBs and neural cells. Interestingly, recent studies have shown that fibroblasts can convert to neurons, underlining the amazing plasticity of these cells [61,62]. 

### 3.7. Overall Impact on Receptors and Integrins

Out of all the DEGs in the hIGFBP5 pFBs, 7% were receptors, including integrins. This is intriguing, since no specific receptor for IGFBP5 has been identified to date. Despite the fact that IGFBP5 does not have a classic integrin binding sequence, it has been shown to bind two ECM proteins that are integrin ligands, THBS1 and SPP1, with high affinity [63,64]. Note that *Thbs1* and *Spp1* were both downregulated in hIGFBP5 pFBs, and seven integrins were DEGs: *Itga2b* and *Itga3* were upregulated and *Itga2*, *Itga7*, *Itga8*, *Itgb5* and *Itgb6* were downregulated. In cancer cells, direct high-affinity interaction between IGFBP5 and α2β1 integrin (aka ITGA2) has been reported [63]. Whether increased to meet the demand of increasing levels of hIGFBP5 or as a negative feedback mechanism to reduce the effects of hIGFBP5 on pFBs, the role of these integrins in the IGFBP5 response warrant further investigation. 

Several GPCRs and adhesion GPCRs were deregulated by hIGFBP5 in pFBs, along with interleukin receptors and prostaglandin receptors, to name a few. The transcriptomic signature we captured in hIGFBP5 pFBs raises the question as to whether IGFBP5 interacts directly with GPCRs or mediates the interaction with other receptors with GPCRs. This is further supported by the functional enrichment analysis which highlighted that many enriched pathways were “receptor interaction” driven and have signal transduction functionalities. 

### 3.8. Downregulation of Bdkrb2, one of the Most Relevant Hub Genes

BDKRB2 is a receptor for bradykinins, small peptides that act as mediators of pain and inflammation, and this receptor is associated with G proteins that activate PI3K-Akt signaling and the calcium second messenger system [65]. Loss of the BDKRB2 receptor in knockout mice induced interstitial fibrosis by decreasing activity of plasminogen activators (PAs) and matrix metalloproteinase-2 (MMP-2) [66]. Interestingly, *Mmp2* was downregulated in hIGFBP5 FBs, along with *Mmp15* and *Mmp16* (only *Mmp10* was upregulated). In contrast, BDKRB2 activation protected against renal fibrosis via the PA/MMP-2 cascade by increasing ECM degradation [66]. Therefore, downregulated levels of *Bdkrb2* could disrupt the urokinase-plasminogen cascade system, hindering its ability to promote ECM degradation, thus resulting in ECM accumulation. Together, this suggests that *Bdrkb2* has anti-fibrotic properties and that its reduced expression by hIGFBP5 may explain, in part, the pro-fibrotic activity of IGFBP5. 

*Bdkrb2* was defined as a hub gene that is involved in six enriched pathways in our functional enrichment analysis, namely neuroactive-ligand receptor interaction, calcium signaling, complement and coagulation cascades, cGMP-PKG signaling, regulation of actin cytoskeleton and endocrine and other factor-regulated calcium reabsorption pathways. In concert with hIGFBP5 downregulation of *Bdkrb2* expression levels in pFBs, our data suggest that restoring *Bdkrb2* levels could have far reaching effects on processes that drive PF. Using DrugBank Online (https://go.drugbank.com/, accessed on 1 November 2021), a comprehensive, free-to-access, online database containing information on drugs and drug targets, we identified Labradimil as an investigational Bdrkb2 agonist (Supplementary Table S6). 

In fact, most of the hub genes identified in this study have chemical, pharmacological or pharmaceutical compounds that target them (Supplementary Table S6). Selective agonists and antagonists for NMUR1 are being developed [67,68]. Anti-CD20 antibody has shown promising results for skin and lung fibrosis by depleting B cells [69], and the humanized monoclonal antibody rituximab effectively reduced cutaneous and pulmonary involvement in SSc patients when combined with mycophenolate mofetil, a reversible inhibitor of inosine monophosphate dehydrogenase [70]. CNR2 agonists WIN55,212 and JWH-133 prevented skin and lung fibrosis in mice injected with hypochlorite, a murine model of SSc [71]. The CXCR4/CXCL12 axis has been shown to enhance the proliferation and migration of human pFBs, leading to the production of collagen; in addition, the antifibrotic effects of CXCR4 antagonists, i.e., AMD3100, have been well characterized in pulmonary studies [72,73,74]. Further, we have shown that IGFBP4, which has anti-fibrotic effects, reduces levels of CXCR4 [56]. The role of PTGER3 in the development of fibrosis is emerging [75,76] and several of its agonists are already listed in the DrugBank (Supplementary Table S6). Niacon, an HCAR2 agonist, has shown anti-fibrotic properties against liver fibrosis in rats [77]. Thus, each of the hub genes identified in this study is targetable by drugs and warrants further examination, as they all have a high degree of centrality and are regulated by IGFBP5. 

### 3.9. Effect of hIGFBP5 on Chemokine and Cytokine Signaling

Chemokines are leukocyte chemoattractants that collaborate with profibrotic cytokines to recruit key effector cells to sites of tissue injury and are important in the development of pulmonary fibrosis and other fibroproliferative disorders [78]. Interestingly, the cytokine–cytokine receptor interaction pathway was enriched in hIGFBP5 pFBs by the differential expression of several chemokines and chemokine receptors known to play a role in the development of fibrosis. For example, CCL2 contributes to the development of PF by inducing IL6 production [79]. The CXCL12/CXCR4 axis, as well as CCL12, are involved in bleomycin-induced PF by regulating the recruitment of fibrocytes to the lung [80,81]. Notably, the expression of *Cxcr4*, *Ccl2* and *Ccl20* was downregulated in hIGFBP5 pFBs, suggesting a possible negative feedback mechanism restricting chemokine receptor signaling. 

### 3.10. Endogenous vs. Exogenous IGFBP5

We generated hIGFBP5-expressing mice because (1) IGFBP5 is well conserved between humans and mice, and (2) we wanted to be able to differentiate the expression levels of endogenous and exogenous IGFBP5. Further, the hIGFBP5 sequence used to generate the mice was cloned from SSc dermal fibroblasts in which IGFBP5 showed significantly increased expression [10]. Compared to previously published data, the effect of hIGFBP5 in pFBs was consistent for *Col1a1*, *Col1a2*, and *Egr1*, as they were all upregulated in pFBs of HOMO mice; however, for *Ctgf*, *Dok5*, *Fn*, *Lox* and *Acta2*, the impact of hIGFBP5 was different than previously reported [11,17,18]. *Ctgf* and *Dok5* were downregulated and *Fn*, *Lox* and *Acta2* were not differentially expressed in transgenic pFBs, although they were increased in whole tissues of the transgenic mice [16]. Our results are most likely due to a unique aspect of IGFBP5, which can exert different effects, whether endogenously expressed or added exogenously as a recombinant protein [82] as well as acute versus chronic expression effects. Further, endogenous and exogenous IGFBP5 might exert different effects in primary pFBs from healthy and diseased cells/tissues [11]. 

### 3.11. Sex Differences in the Response of Fibroblasts

Our data show that gene expression levels in WT mice and in response to transgene expression may differ based on sex. Sex differences in the response of C57BL/6 mice to experimental bleomycin induced lung fibrosis have been reported, with male mice exhibiting more severe disease than female mice. Female mice given exogenous androgens exhibited responses similar to those of WT males [83]. This is in contrast to rats, where female mice challenged with endotracheal bleomycin had more severe lung fibrosis and higher tissue levels of collagen I and Tgfβ1 than male rats, and the extent of fibrosis was reduced when female rats were ovariectomized [84]. 

Differential sex-based effects of Igfbp5 were described for transgenic mice expressing Igfbp5 under the control of the β-actin promoter, where differential effects on bone mineral density were observed in male mice when compared to female mice, with higher serum levels of Igfbp5 noted in males [85]. Several studies have suggested that IGFBP5 can mediate the effects of sex steroids, including estrogen [86,87,88,89]. We have previously shown that estradiol levels are elevated in post-menopausal women with SSc and estradiol induces a fibrotic phenotype in human skin [90,91]. These findings suggest that sex hormones, and specifically estrogen, can promote a more fibrotic milieu in females. Thus, differences in gene expression in female and male hIGFBP5 transgenic mice in our study may be due to the potential downstream effects of sex hormones. 

Our recent data suggest that hIGFBP5 transgene mRNA expression levels in lung tissues of male and female HOMO mice are not significantly different [16]. However, our current results suggest that pFBs isolated from lung tissues of transgenic female mice have higher levels of secreted hIGFBP5 protein. The hIGFBP5 transgene expression is regulated via the elongation factor 1 a1 (Ef1a1) promoter [16]. There is no data suggesting that transgene levels expressed under the control of the Ef1a1 promoter are regulated differently in male versus female mice. In fact, Ef1a1 is often used as a ‘housekeeping’ or reference gene as a result of its stable expression. However, two studies showed that Ef1a1 expression can be increased by estradiol in liver tissues of female tadpoles [92] and in neurons in the hypothalamic anteroventral periventricular region of mice [93]. This suggests that higher levels of Igfbp5 protein secreted by pFBs from transgenic female mice in our study may be due to the effect of estrogen, resulting in more pronounced differential expression of downstream target genes. This is conceptually not surprising, as Oliva et al. have shown that 37% of genes exhibit sex-based expression in a tissue-specific manner [94]. 

### 3.12. Conclusions

Expression of hIGFBP5 in mice allowed us to perform a full functional enrichment analysis and identify impacted pathways and hub genes that could be potential mediators and markers of PF. Ligand-receptor interactions were strongly affected by hIGFBP5, and 7% of the DEGs were differentially expressed receptors, including integrins. The ECM was also majorly perturbed by hIGFBP5, along with fundamental pathways that contribute to fibrogenesis. This study is a steppingstone for future validation experiments and identification of potential therapeutic targets as it provides new insights into the molecular mechanisms of IGFBP5-induced PF. 

## 4. Materials and Methods

### 4.1. Ethics Statement

All the animal experiments were approved by the Medical University of South Carolina (MUSC, Charleston, SC, USA) Institutional Animal Care and Use Committee (IACUC). 

### 4.2. Culture of Primary Mouse Lung Fibroblasts

Primary fibroblasts from lung tissues of mice were cultured in Dulbecco’s modified Eagle’s medium (DMEM) (Mediatech, Inc., Manassas, VA, USA) supplemented with 10% fetal bovine serum (FBS) (Sigma-Aldrich, St. Louis, MO, USA), penicillin, streptomycin, and antimycotic agent (Invitrogen, Carlsbad, CA, USA), as previously described [12,55]. Briefly, the lung tissues were minced, and pieces were allowed to adhere to 100-mm tissue culture dishes for one hour prior to the addition of culture medium. Fibroblasts were cultured at 37 °C with 5% CO_2_ in a humidified atmosphere. For experiments, 2.0 × 10^5^ primary fibroblasts were plated in each well of 6-well tissue culture plates in 10% FBS-containing DMEM. After 96 h, conditioned media and cellular lysates were harvested. Cells were used in passages 3 to 7. 

### 4.3. RNA Extraction and Preparation

Total RNA was extracted from pFBs using TRIzol (Invitrogen, Carlsbad, CA, USA) per the manufacturer’s instructions, and RNA quality and quantity were assessed using a NanoDrop Lite spectrophotometer (ThermoFisher, Waltham, MA, USA). 

### 4.4. RNA Sequencing & Differential Expression Analysis

Total RNA from pFBs of female mice were sent to Novogene Corporation Inc. (Sacramento, CA, USA) for RNAseq analysis. RNA degradation and contamination were monitored on 1% agarose gels, RNA purity was checked using the NanoPhotometer^®^ spectrophotometer (IMPLEN, Los Angeles, CA, USA), and RNA integrity and quantitation were assessed using the RNA Nano 6000 Assay Kit of the Bioanalyzer 2100 system (Agilent Technologies, Santa Clara, CA, USA). A total amount of 1 μg RNA per sample was used as input material for the RNA sample preparations. Sequencing libraries were generated using NEBNext^®^ UltraTM RNA Library Prep Kit for Illumina^®^ (NEB, Ipswich, MA, USA) following the manufacturer’s recommendations; index codes were added to attribute sequences to each sample. Briefly, mRNA was purified from total RNA using poly-T oligo-attached magnetic beads. Fragmentation was carried out using divalent cations under elevated temperature in NEBNext First Strand Synthesis Reaction Buffer (5X). First strand cDNA was synthesized using random hexamer primer and M-MuLV Reverse Transcriptase (RNase H-). Second strand cDNA synthesis was subsequently performed using DNA Polymerase I and RNase H. Remaining overhangs were converted into blunt ends via exonuclease/polymerase activities. After adenylation of 3′ ends of DNA fragments, an NEBNext Adaptor with hairpin loop structure was ligated to prepare for hybridization. To select cDNA fragments of preferentially 150~200 bp in length, the library fragments were purified with an AMPure XP system (Beckman Coulter, Brea, CA, USA). Then 3 μL USER Enzyme (NEB, USA) was used with size-selected, adaptor-ligated cDNA at 37 °C for 15 min, followed by 5 min at 95 °C before PCR. Next, PCR was performed with Phusion High-Fidelity DNA polymerase, Universal PCR primers and Index (X) Primer. Finally, PCR products were purified (AMPure XP system) and library quality was assessed on the Agilent Bioanalyzer 2100 system. The clustering of the index-coded samples was performed on a cBot cluster generation system using PE Cluster Kit cBot-HS (Illumina) according to the manufacturer’s instructions. After cluster generation, the library preparations were sequenced on an Illumina platform and paired-end reads were generated on the Illumina NovaSeq 6000 instrument (Illumina, San Diego, CA, USA). 

Raw data (raw reads) of FASTQ format were firstly processed through fastp. In this step, clean data (clean reads) were obtained by removing reads containing adapter and poly-N sequences and reads with low quality from raw data. At the same time, Q20, Q30 and GC content of the clean data were calculated. All the downstream analyses were based on the clean data with high quality. Paired-end clean reads were aligned to the mm10 reference genome using the Spliced Transcripts Alignment to Reference (STAR) software. FeatureCounts was used to count the read numbers mapped of each gene. “HOMO vs. WT” was carried out by DESeq2 [95]. For each gene, DESeq2 reported estimated log2 fold change (log2FC) and provided a false discovery rate (FDR)-adjusted *p*-value (*q*-value). Transcript count data were sorted according to their *q*-value. FDR is the expected fraction of false positive tests among significant tests and was calculated using the Benjamini-Hochberg multiple testing adjustment procedure. Differentially expressed genes (DEGs) were defined by *q*-value < 0.05 and log2FC > 0.6 for upregulated DEGs, and log2FC < −0.6 for downregulated DEGs [96,97]. 

### 4.5. Functional Enrichment & Hub Gene Network Analysis

The DEGs generated by DESeq2 in the comparison “HOMO vs. WT” were imported into iPathwayGuide, which provides an *impact analysis method* [98,99,100] that quickly identifies the significantly impacted pathways based on two forms of evidence: Over representation analysis and accumulation analysis (iPathwayGuide by Advaita https://advaitabio.com/ipathwayguide, accessed on 1 November 2021, Ann Arbor, MI, USA). Over-representation calculates a probability (pORA) of a gene set impacting a given pathway based on the number of DEGs in the gene set mapped to that pathway compared to the number of DE genes expected just by chance [98]. *Accumulation* computes a probability (pAcc) by considering the measured fold change of all DE genes on a pathway, the position and role of each DEG, as well as the direction and type of all the signals in the pathway. A perturbation factor is calculated for gene A based on the fold change of gene B upstream of gene A in the pathway, as well as the type of regulation that gene B exhibits on gene A. The probabilities pORA and pAcc are then combined in a pathway-level *p*-value. The underlying pathway topologies, comprised of genes and their directional interactions, are obtained from the KEGG database. The impact analysis has been shown to be vastly superior to the simple enrichment analysis [101]. 

The *perturbation* calculated for each gene also allows the identification of putative mechanisms. These appear as cascades of coherent perturbation propagation. An edge in a pathway is coherent if the measured changes are consistent with the phenomena described by the pathway. For instance, if a gene A is known to inhibit a gene B immediately downstream of it and if gene A has been measured to be up-regulated, while B has been measured to be down-regulated, that edge would be coherent. A continuous sequence of several coherent edges constitutes a coherent cascade of perturbation propagation. 

iPathwayGuide relies on the Kyoto Encyclopedia of Genes and Genomes (KEGG) database (Release 96.0+/11–21, 20 November) [102,103] as well as the Gene Ontology Consortium database (14 October 2020) to annotate genes and analyze the biological processes (BPs) of the genes and include the BP, cellular component (CC) and molecular function (MF) [104,105]. A *p*-value < 0.05 was set to indicate significant difference. iPathwayGuide uses two proprietary approaches for gene ontology (GO) analysis. These approaches are focused on removing the intrinsic GO redundancy and improving the analysis accuracy. The *high-specificity pruning* (highSP) approach calculates *p*-values for GO terms using a hypergeometric approach starting at the lowest level of GO’s directed acyclic graph, with the more specific GO terms first, and proceeding up the GO hierarchy through the more general terms. For lower-level GO terms that are not found significant, the DEGs in those terms will be propagated up through the GO directed acyclic graph. Once a GO term in the hierarchy is found significant, the propagation stops. *Smallest common denominator pruning* (SCDP) identifies and reports GO terms that are parent terms of multiple more-specific GO terms calculated to be significant. 

iPathwayGuide calculates a gene network based on gene interaction data obtained from STRING and BioGRID. Hub genes are determined by ranking genes by the total number of interactions with other genes in the network; nodes with the largest number of incoming edges are found at the center, and those with the fewest are on the periphery, forming a “wheel hub” network. The degree of centrality for each node is reported in a table, with “1” representing the highest degree of connectivity, and “0” the lowest. The top 500 DEGs with the highest absolute value of log2FC were used to construct the network (Supplementary Table S5). It has been previously shown that proteins that play an essential role and are biologically relevant have a mean centrality value that is significantly higher than the centrality value of nonessential proteins [106]. Therefore, we focused on genes with a degree of centrality ≥0.5. 

ToppFun from ToppGene Suite [107] was also utilized to obtain a functional enrichment analysis of the differentially expressed receptors and integrins identified amongst all the DEGs in hIGFFBP5 pFBs. 

### 4.6. Quantitative Reverse Transcription (qRT-PCR)

First-strand cDNA was reverse-transcribed with an oligo (dT)12-15 primer (Invitrogen, Carlsbad, CA, USA) and SuperScript IV Reverse Transcriptase (Invitrogen), and cDNA was used for qRT-PCR. Gene mRNA expression levels were measured using the TaqMan^®^ real-time PCR system (Applied Biosystems, Foster City, CA, USA) according to the manufacturer’s protocol. Expression levels were normalized to beta-2-microglobulin (*B2m*) (Mm00437762_m1). Relative expression levels were compared using the comparative CT method formula 2^−ΔΔCt^. Specific primers and probes for amplifying genes encoding mouse interleulin-6, *Il-6* (Mm00446190_m1), mouse collagen type XXVII alpha 1, *Col27a1* (Mm00508542_m1), mouse procollagen C-endopeptidase enhancer 2, *Pcolce2* (Mm00453052_m1), mouse bradykinin receptor beta 2, *Bdkrb2* (Mm00437788_s1), mouse neuromedin U receptor 1, *Nmur1* (Mm00515885_m1), mouse caveolin 3, *Cav3* (Mm00725536_s1), and mouse secreted phosphoprotein, *Spp1* (Mm00436767_m1) were used. Mouse *Gapdh* (Mm99999915_g1) was also used to confirm results obtained with *B2m* with no notable differences (data not shown). 

### 4.7. Immunoblotting

Conditioned media and cell lysates from cultured primary pFBs were analyzed by immunoblotting. Equal amounts of protein were separated by electrophoresis using 10% SDS-PAGE gels in 1× SDS running buffer (25 mM Tris, 250 mM Glycine and 0.1% SDS) at 100 Volts for 1.5 h and transferred to nitrocellulose membranes (Amersham, Marlborough, MA, USA) in Western transfer buffer (25 mM Tris, 192 mM Glycine and 20% MeOH) at 0.3 milli-Amps (mAmp) for 2 h on ice. Membranes were incubated with primary antibody. The following antibodies were used: human IGFBP5 polyclonal antibody (GroPep Bioreagents, Thebarton, SA, Australia), IGFBP-4 (clone C-20) monoclonal (Santa Cruz, Dallas, TX, USA), plasminogen activator inhibitor type 1 (PAI-1 aka Serpine1) (clone 1H4A5) monoclonal antibody (Proteintech, Rosemont, IL, USA), secreted phosphoprotein 1 (SPP-1) monoclonal antibody (Epitomics, Inc., Burlingame, CA, USA), T-box transcription factor 1 (TBX1) (clone OTI1C2) monoclonal antibody (Novus Biologicals, Littleton, CO, USA), neuromedin U receptor 1 (NMUR1) polyclonal antibody, (Novus Biologicals), bradykinin receptor beta 2 (BDKRB2) polyclonal antibody (Novus Biologicals), formyl peptide receptor 1 (FRP1) polyclonal antibody (Novus Biologicals), pappalysin-2 (PAPPA2) polyclonal antibody (Novus Biologicals), transforming growth factor beta 2 (TGFB2) (Clone ab36495) monoclonal antibody (Abcam, Cambridge, UK), and beta-actin (β-actin) monoclonal antibody (Santa Cruz) as primary antibodies and horseradish peroxidase-conjugated antibody as a secondary antibody. Signals were detected using chemiluminescence on FluorChem R System (ProteinSimple, San Jose, CA, USA). Densitometry was analyzed with ImageJ software (U.S. National Institutes of Health, Bethesda, MD, USA). 

### 4.8. Ponceau S Staining

After the transfer, membranes were stained with Ponceau S staining solution (0.1% (*w*/*v*) Ponceau S in 5% (*v*/*v*) acetic acid) for 1 h at room temperature with rotation. The membrane was washed 3 times with distilled water until the background was clear and imaged on FluorChem R System (ProteinSimple, San Jose, CA, USA). 

### 4.9. Statistical Analysis

All continuous variables were expressed as the mean ± standard deviation. All statistical analyses were done using GraphPad Prism version 8 for Windows (GraphPad Software, La Jolla, CA, USA). Unpaired *t*-test was used for comparison between WT and HOMO mouse groups. All *p*-values < 0.05 were considered statistically significant. 

## Figures and Tables

**Table 1:** Functional enrichment analysis. The enrichment analysis performed on iPathwayGuide includes gene ontology (GO) and pathway (PW) analyses. GO analysis was performed using 2 corrections: the high specificity pruning (highSP) and the smallest common denominator pruning (SCDP) corrections. BP: biological process, MF: molecular function, CC: cellular component. The impacted pathway analysis was performed without any correction: PW: pathway. Only the top 10 most enriched pathways are listed here. For a full list, see Supplementary Table S2. Yellow: Angiogenesis related terms, orange: MAPK signaling and downstream pathways affected by it, green: calcium ion binding, pink: components of the plasma membrane including receptor and ligand activity, blue: ECM-related terms.

Category	Name	Count DEG	Count All	*p*-Value	Correction
BP	Angiogenesis	134	446	3.67E-04	SCDP
BP	positive regulation of MAPK cascade	117	412	8.15E-03	SCDP
BP	regulation of blood coagulation	28	62	1.32E-02	SCDP
BP	regulation of cell migration	217	798	2.55E-02	SCDP
BP	morphogenesis of a branching structure	70	199	3.05E-02	SCDP
BP	positive regulation of cell population proliferation	190	764	4.07E-02	SCDP
BP	biological adhesion	287	1046	4.30E-02	SCDP
BP	Ossification	88	312	4.30E-02	SCDP
BP	response to gonadotropin	8	10	4.30E-02	SCDP
MF	calcium ion binding	111	411	1.71E-02	highSP
MF	cytokine activity	39	110	1.71E-02	highSP
MF	growth factor activity	33	91	2.96E-02	highSP
MF	receptor ligand activity	81	246	5.69E-05	SCDP
MF	calcium ion binding	111	411	8.65E-03	SCDP
MF	carbohydrate binding	58	185	9.67E-03	SCDP
MF	cell adhesion molecule binding	60	195	9.67E-03	SCDP
MF	extracellular matrix structural constituent	39	117	3.03E-02	SCDP
MF	glycosaminoglycan binding	48	154	3.03E-02	SCDP
CC	extracellular space	271	867	9.14E-17	highSP
CC	integral component of plasma membrane	231	819	4.75E-08	highSP
CC	cell surface	189	625	9.10E-08	highSP
CC	extracellular matrix	122	377	1.04E-05	highSP
CC	integral component of membrane	749	3218	3.56E-05	highSP
CC	collagen-containing extracellular matrix	85	293	2.37E-03	highSP
CC	external side of plasma membrane	75	266	2.37E-02	highSP
CC	Z disc	32	95	3.71E-02	highSP
CC	extracellular region	395	1362	7.12E-18	SCDP
CC	intrinsic component of membrane	770	3318	1.96E-12	SCDP
CC	cell surface	189	625	4.75E-11	SCDP
CC	apical part of cell	91	318	1.25E-03	SCDP
CC	I band	35	103	2.61E-02	SCDP
PW	Cytokine–cytokine receptor interaction	52	163	6.45E-07	none
PW	Focal adhesion	47	187	2.46E-05	none
PW	Staphylococcus aureus infection	14	37	3.04E-05	none
PW	Viral protein interaction with cytokine and cytokine receptor	18	50	6.95E-05	none
PW	AGE-RAGE signaling pathway in diabetic complications	34	98	1.29E-04	none
PW	Systemic lupus erythematosus	20	77	1.97E-04	none
PW	Proteoglycans in cancer	54	186	2.79E-04	none
PW	Chemokine signaling pathway	35	147	7.48E-04	none
PW	Neuroactive ligand-receptor interaction	38	133	1.10E-03	none
PW	Calcium signaling pathway	34	150	2.06E-03	none

**Table 2:** Enriched pathways containing hub genes. The 12 hub genes are shown here in the context of enriched pathways they belong to. The table also provides the *p*-value of each pathway as well as the biological outcomes of each pathway. Red: upregulation. Blue: downregulation.

Pathways	Genes	*p*-Value	Outcomes
Cytokine–cytokine receptor interaction	*Cxcr4*, *Ccl20*, *Ccl28*	6.45E-07	Innate and adaptive inflammatory host defenses, cell growth, differentiation, cell death, angiogenesis, development and repair processes aimed at the restoration of homeostasis
Chemokine signaling pathway	*Cxcr4*, *Ccl20*, *Ccl28*	7.48E-04	Inflammatory immune response, cellular activation, differentiation and survival, cellular polarization and actin reorganization
Neuroactive ligand-receptor interaction	*Fpr1*, *Bdkrb2*, *Mchr1*, *Nmur1*, *Cnr2*, *P2ry14*, *Ptger3*	1.10E-03	Environmental information processing
Calcium signaling pathway	*Gna15*, *Bdkrb2*, *Cxcr4*, *Ptger3*	2.06E-03	MAPK signaling, apoptosis, long-term potentiation/depression, phosphatidylinositol signaling pathway, contraction, metabolism, proliferation
Axon guidance	*Cxcr4*	2.15E-03	Formation of neuronal network, cytoskeletal organization
Complement and coagulation cascades	*Bdkrb2*	4.48E-03	Innate immunity, recruitment of inflammatory and immunocompetent cells
cGMP-PKG signaling pathway	*Bdkrb2*	1.98E-02	Physiologic processes, regulation of cytosolic calcium concentration and sensitivity of myofilaments to Ca^2+^, ROS release from mitochondria
Regulation of actin cytoskeleton	*Bdkrb2*	2.92E-02	Focal adhesion, MAPK signaling, adherens junction,
Endocrine and other factor-regulated calcium reabsorption	*Bdkrb2*	4.33E-02	Intracellular signalling processes, neuronal excitability, muscle contraction and bone formation
Rap1 signaling pathway	*Fpr1*	5.01E-03	Cell adhesion, cell–cell junction formation and cell polarity, control of cellcell and cell-matrix interactions by regulating the function of integrins and other adhesion molecules, MAPK signaling
TNF signaling pathway	*Ccl20*	2.71E-02	Apoptosis and cell survival as well as inflammation and immunity, MAPK cascade, apoptosis, necroptosis, PI3K-dependent NF-kappa B pathway, JNK pathway, survival.
cAMP signaling pathway	*Hcar2*, *Ptger3*	0.487	Metabolism, secretion, calcium homeostasis, muscle contraction, cell fate, and gene transcription

**Table 3:** Summary of experiments. Selected GOIs that were DEGs in hIGFBP5 pFBs of female HOMO mice were further analyzed by qRT-PCR and immunoblotting (cell lysates and conditioned media abbreviated cond. media). Down (downregulated, blue) and Up (upregulated, red) indicates the direction of regulation in hIGFBP5 pFBs of HOMO female mice as compared to pFBs of WT female mice. NA: not analyzed.

Genes	Full Name	RNAseq (F)		qRT-PCR	Immunoblot Cell Lysates	Immunoblot Cond. Media
		Log2Fc	*q*-Value	Regulation	Regulation	Regulation
*Col27a1*	collagen, type XXVII, alpha 1	3.201	1.00E-06	Up	NA	NA
*Pcolce2*	procollagen C-endopeptidase enhancer 2	3.124	1.12E-03	Up	NA	NA
*Pappa2*	pappalysin 2	1.351	2.00E-03	NA	NA	Up
*Nmur1*	neuromedin U receptor 1	−3.449	4.61E-02	Down	Down	NA
*Il6*	interleukin 6	−3.021	1.00E-06	Down	NA	NA
*Bdkrb2*	bradykinin receptor, beta 2	−5.230	5.77E-04	Down	Down	NA
*Cav3*	caveolin 3	−7.164	1.00E-06	Down	NA	NA
*Spp1*	secreted phosphoprotein 1	−3.034	2.21E-02	Down	Down	NA
*Serpine1*	serpin family E member 1	−2.515	1.00E-06	NA	Down	Down
*Fpr1*	formyl peptide receptor 1	−5.713	7.96E-03	NA	Down	NA
*Tbx1*	T-box 1	−9.079	4.20E-02	NA	Down	NA
*Tgfβ2*	transforming growth factor, beta 2	−1.178	1.00E-06	NA	Down	NA
*Igfbp4*	insulin-like growth factor binding protein 4	−3.539	1.00E-06	NA	NA	Down

## Data Availability

The data presented in this study are openly available in the NCBI Gene Expression Omnibus (GEO) at https://www.ncbi.nlm.nih.gov/geo/, accession number GSE184387.

## Supplementary Materials

The following are available online at https://www.mdpi.com/article/10.3390/ijms222212609/s1.
