# Supplementary material for: Identification of Impacted Pathways and Transcriptomic Markers as Potential Mediators of Pulmonary Fibrosis in Transgenic Mice Expressing Human IGFBP5
Source: Int J Mol Sci. 2021 Nov 22;22(22):12609. doi: 10.3390/ijms222212609 (PMC8619832; doi:10.3390/ijms222212609)

**Figure S1: AGE-RAGE signaling pathway in diabetic complications**. **(A)** DEGs in hIGFBP5 fibroblasts that belong to this pathway. **(B)** KEGG pathway showing the logFC of DEGs in (A). Red: upregulated, blue: downregulated, yellow arrow and box highlight biological outcomes.

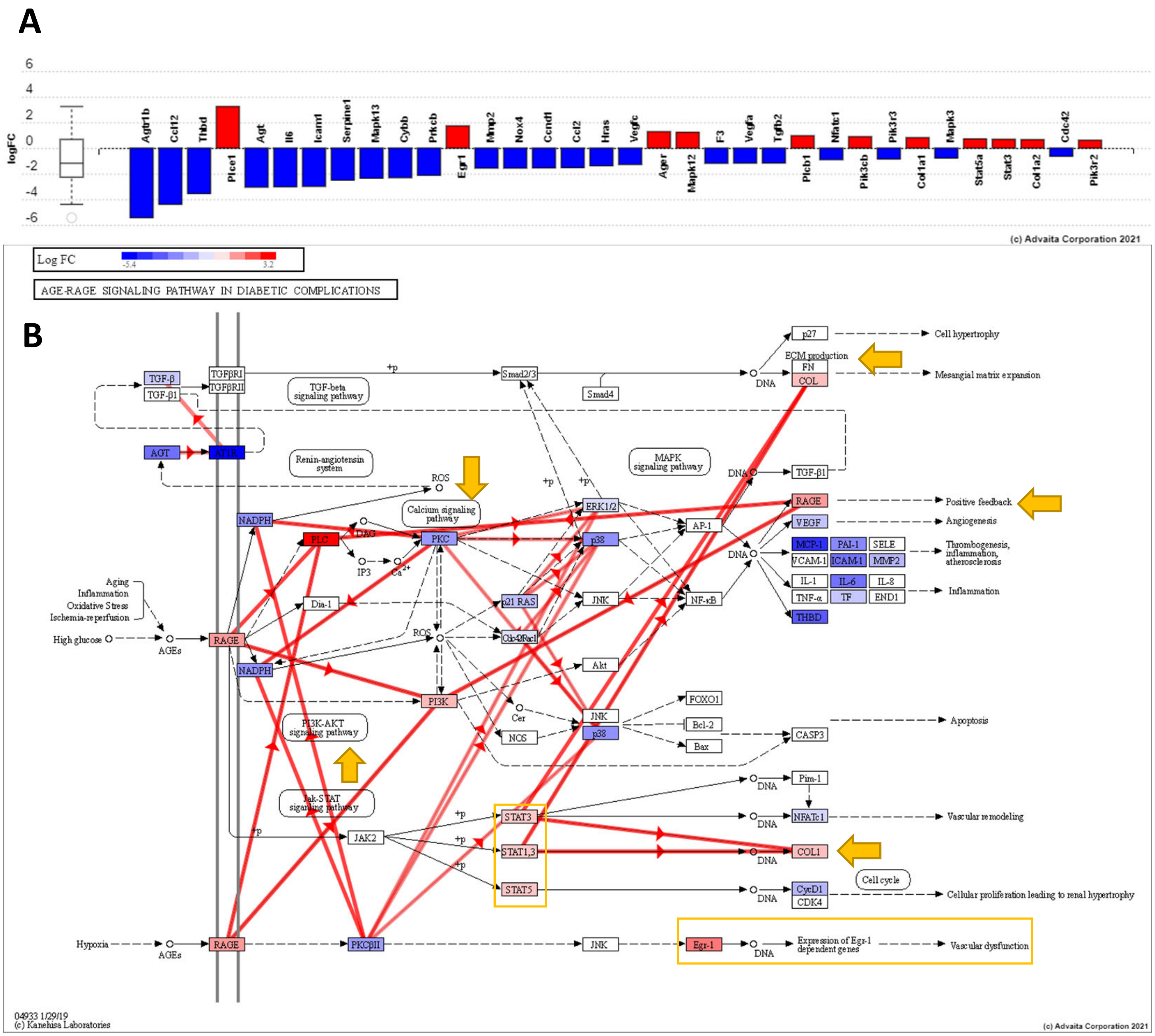

**Figure S2: ECM receptor interaction. (A)** DEGs in hIGFBP5 fibroblasts that belong to this pathway. **(B)** KEGG pathway showing the logFC of DEGs in (A). Red: upregulated, blue: downregulated.

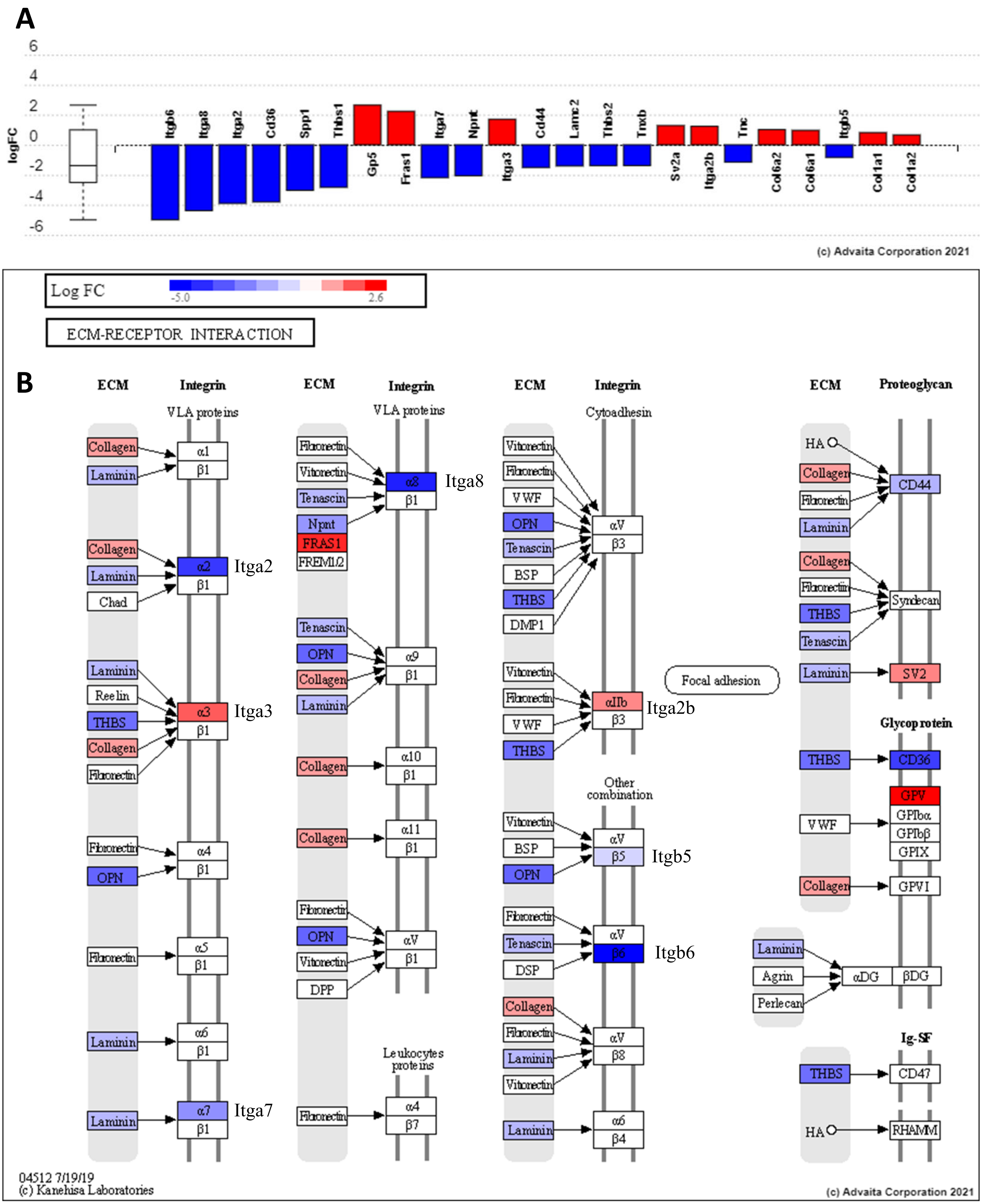

**Figure S3: Focal adhesion. (A)** DEGs in hIGFBP5 fibroblasts that belong to this pathway. **(B)** KEGG pathway showing the predicted perturbation of the transcriptomic signature in (A) in hIGFBP5 fibroblasts. Red: upregulated, blue: downregulated, yellow arrow and box highlight biological outcomes.

**A**

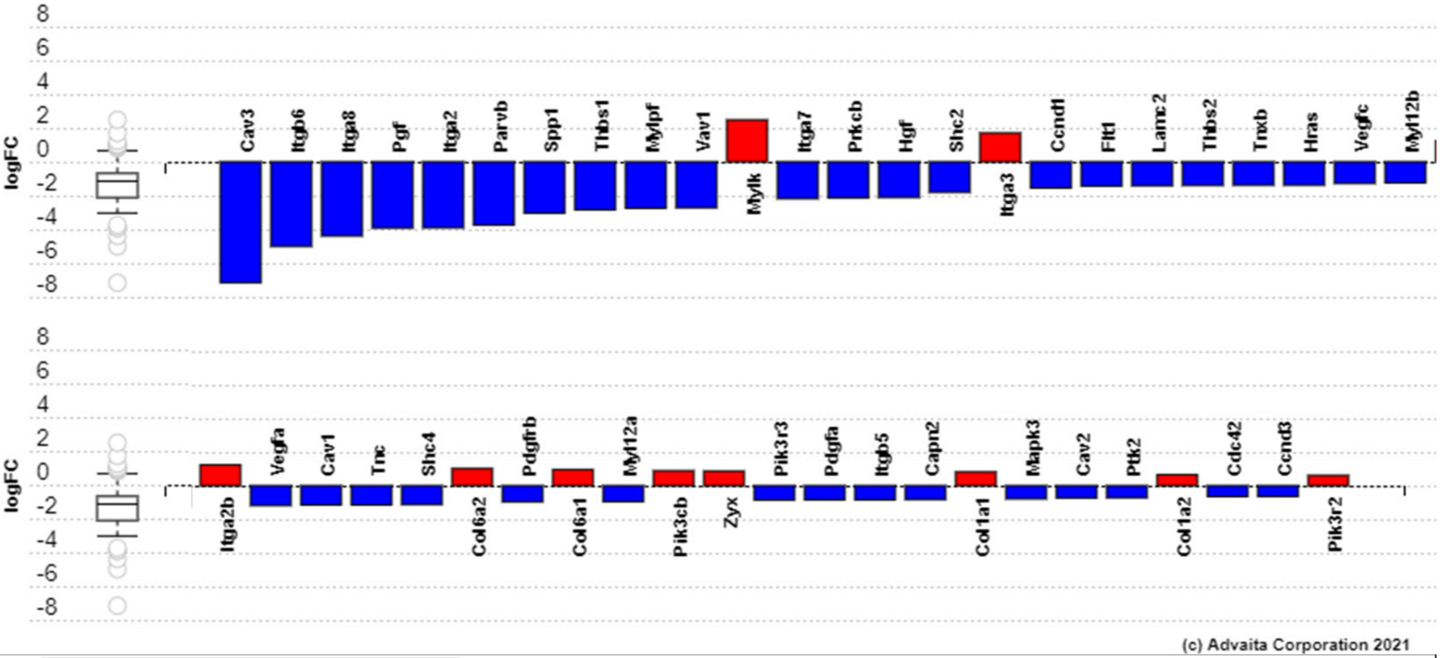


**B**

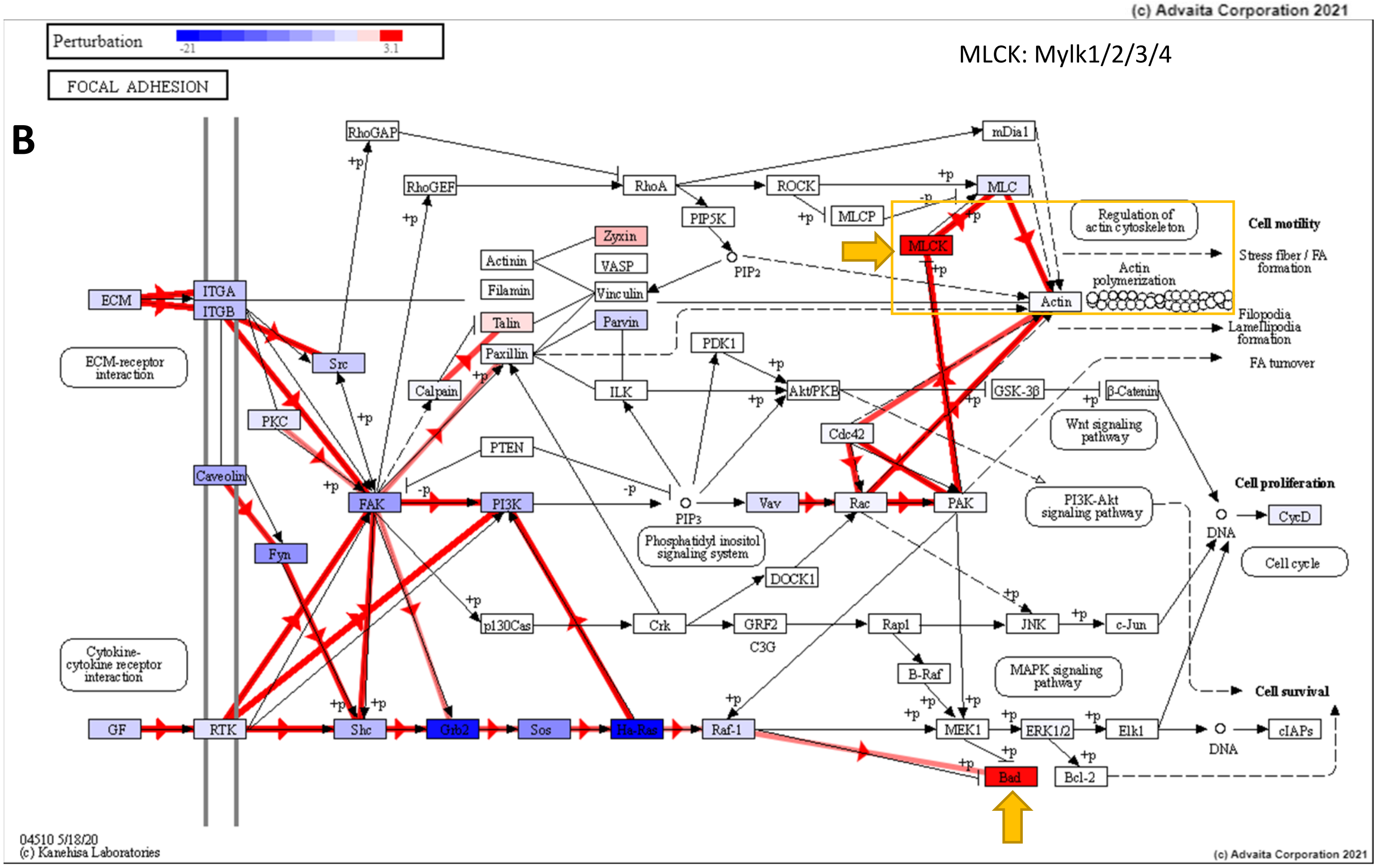

**Figure S4: Regulation of actin cytoskeleton. (A)** DEGs in hIGFBP5 fibroblasts that belong to this pathway. **(B)** KEGG pathway showing the predicted accumulation of the transcriptomic signature in (A) in hIGFBP5 fibroblasts. Red: upregulated, blue: downregulated, green: hub gene(s). **(C)** Barplot of the logFC of hub gene(s).

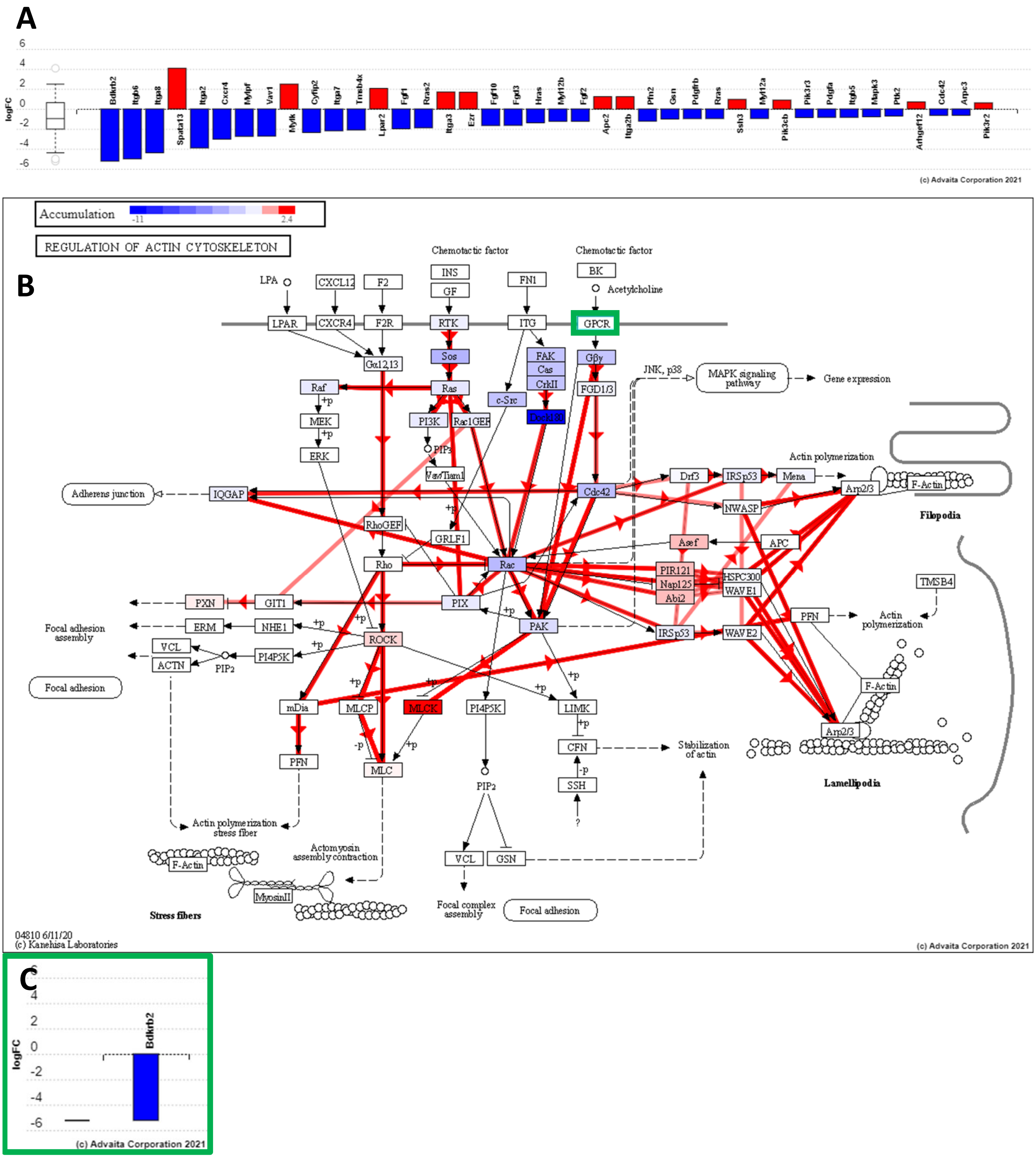

**Figure S5: Cytokine-cytokine receptor interaction. (A)** DEGs in hIGFBP5 fibroblasts that belong to this pathway. **(B)** KEGG pathway showing the predicted accumulation of the transcriptomic signature in (A) in hIGFBP5 fibroblasts. Red: upregulated, blue: downregulated, yellow box highlights IL1-like cytokine receptors in the accumulation KEGG pathway, green: hub gene(s). **(C)** Barplot of the logFC of hub gene(s).

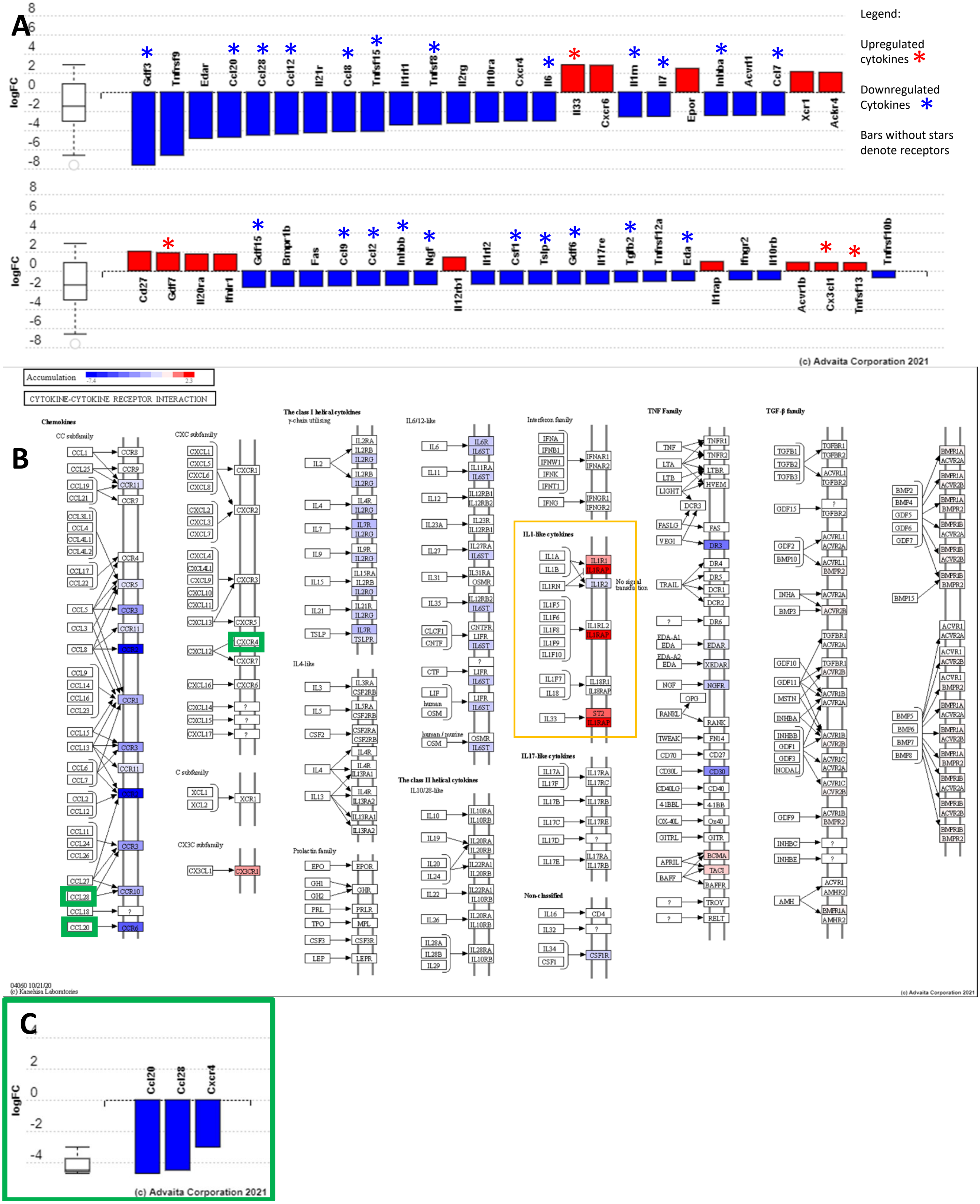

**Figure S6: PI3K-Akt signaling pathway. (A)** DEGs in hIGFBP5 fibroblasts that belong to this pathway. **(B)** KEGG pathway showing the predicted accumulation of the transcriptomic signature in (A) in hIGFBP5 fibroblasts. Red: upregulated, blue: downregulated, yellow arrow and box highlight major ligand/receptor interactions enriched in this pathway and biological outcomes.

# A

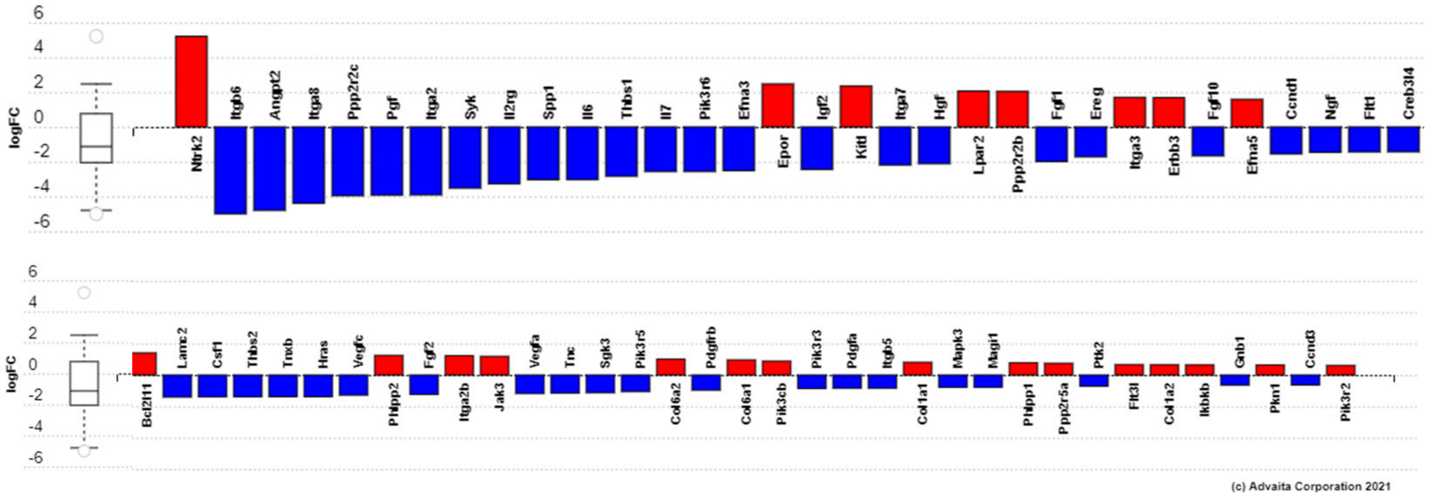


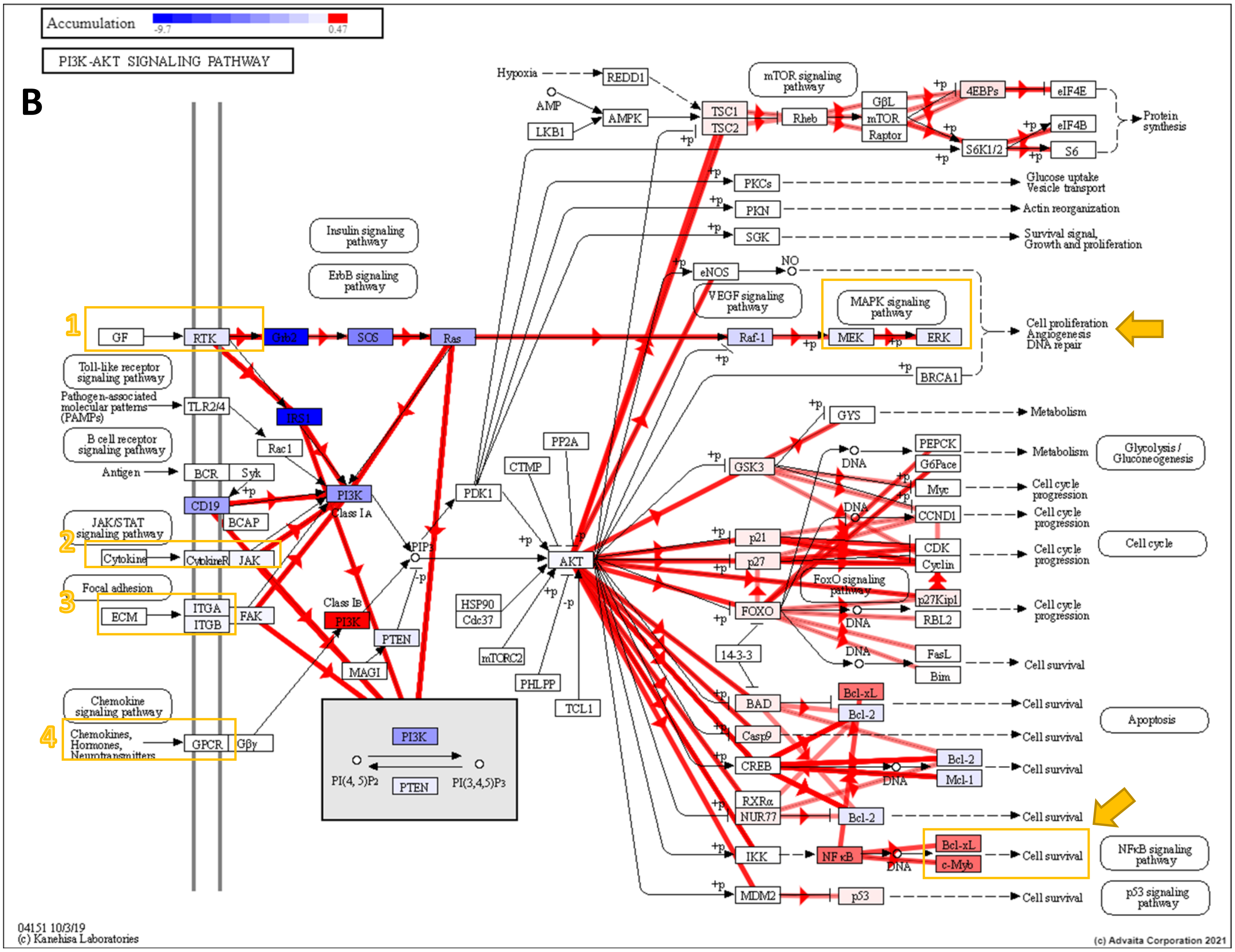

**Figure S7: Chemokine signaling pathway. (A)** DEGs in hIGFBP5 fibroblasts that belong to this pathway. **(B)** KEGG pathway showing the predicted perturbation of the transcriptomic signature in (A) in hIGFBP5 fibroblasts. Red: upregulated, blue: downregulated, green: hub gene(s). **(C)** Barplot of the logFC of hub gene(s).

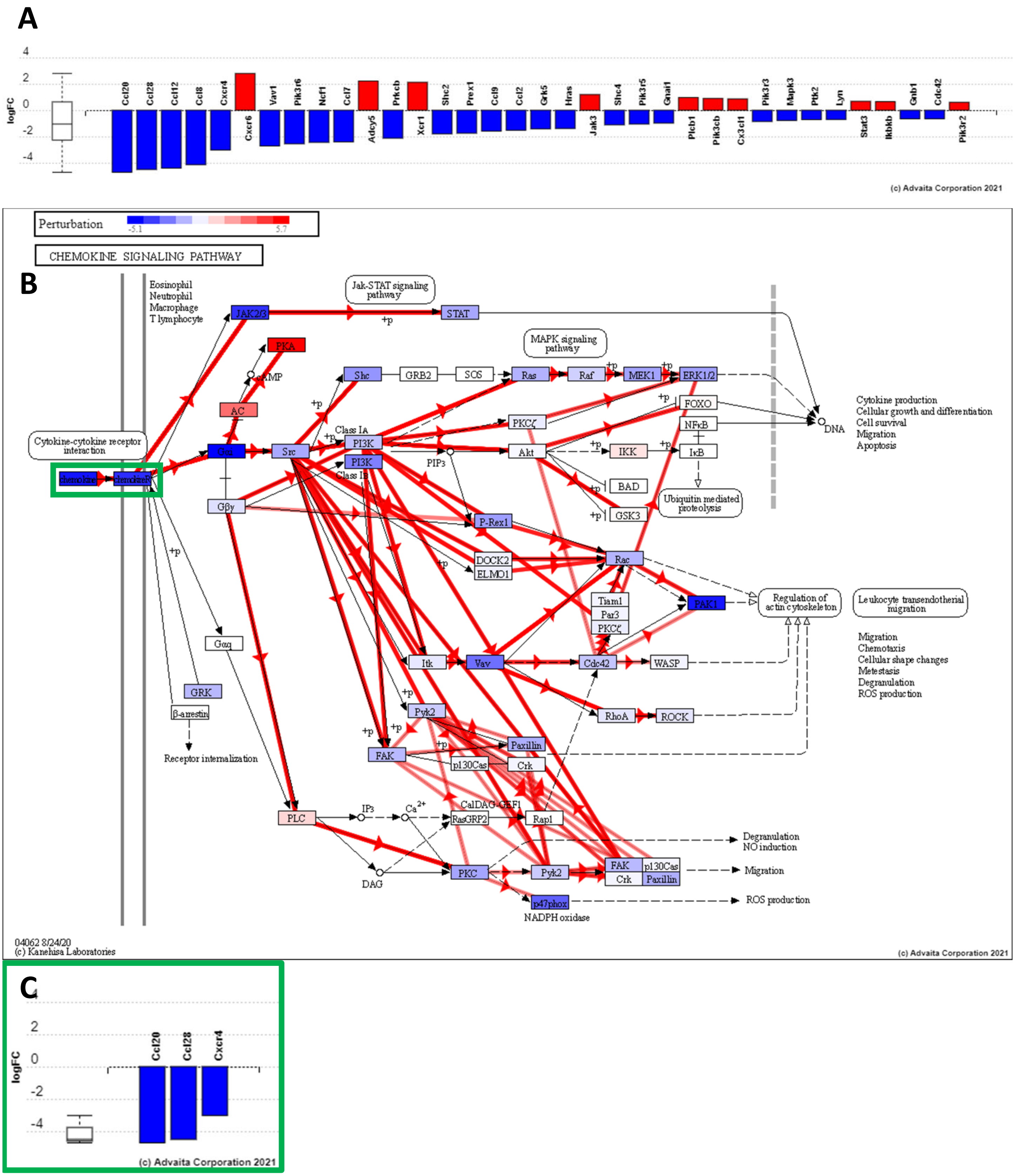

**Figure S8: MAPK signaling pathway**. **(A)** DEGs in hIGFBP5 fibroblasts that belong to this pathway. **(B)** KEGG pathway showing the predicted perturbation of the transcriptomic signature in (A) in hIGFBP5 fibroblasts. Red: upregulated, blue: downregulated.

A

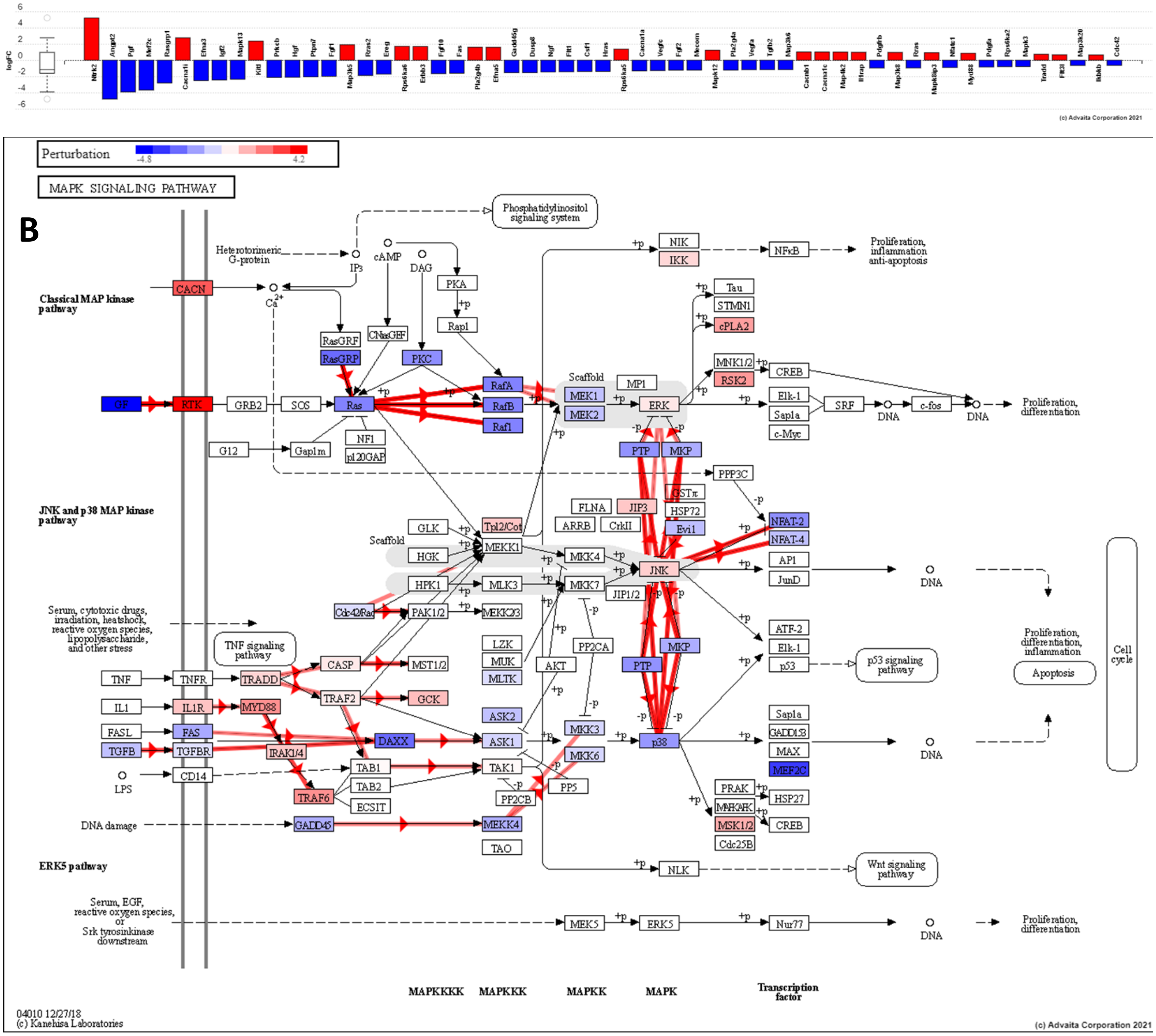

**Figure S9: Calcium signaling pathway. (A)** DEGs in hIGFBP5 fibroblasts that belong to this pathway. **(B)** KEGG pathway showing the logFC of DEGs in (A). Red: upregulated, blue: downregulated, yellow boxes highlight neurotransmitter ligands, green: hub gene(s). **(C)** Barplot of the logFC of hub gene(s).

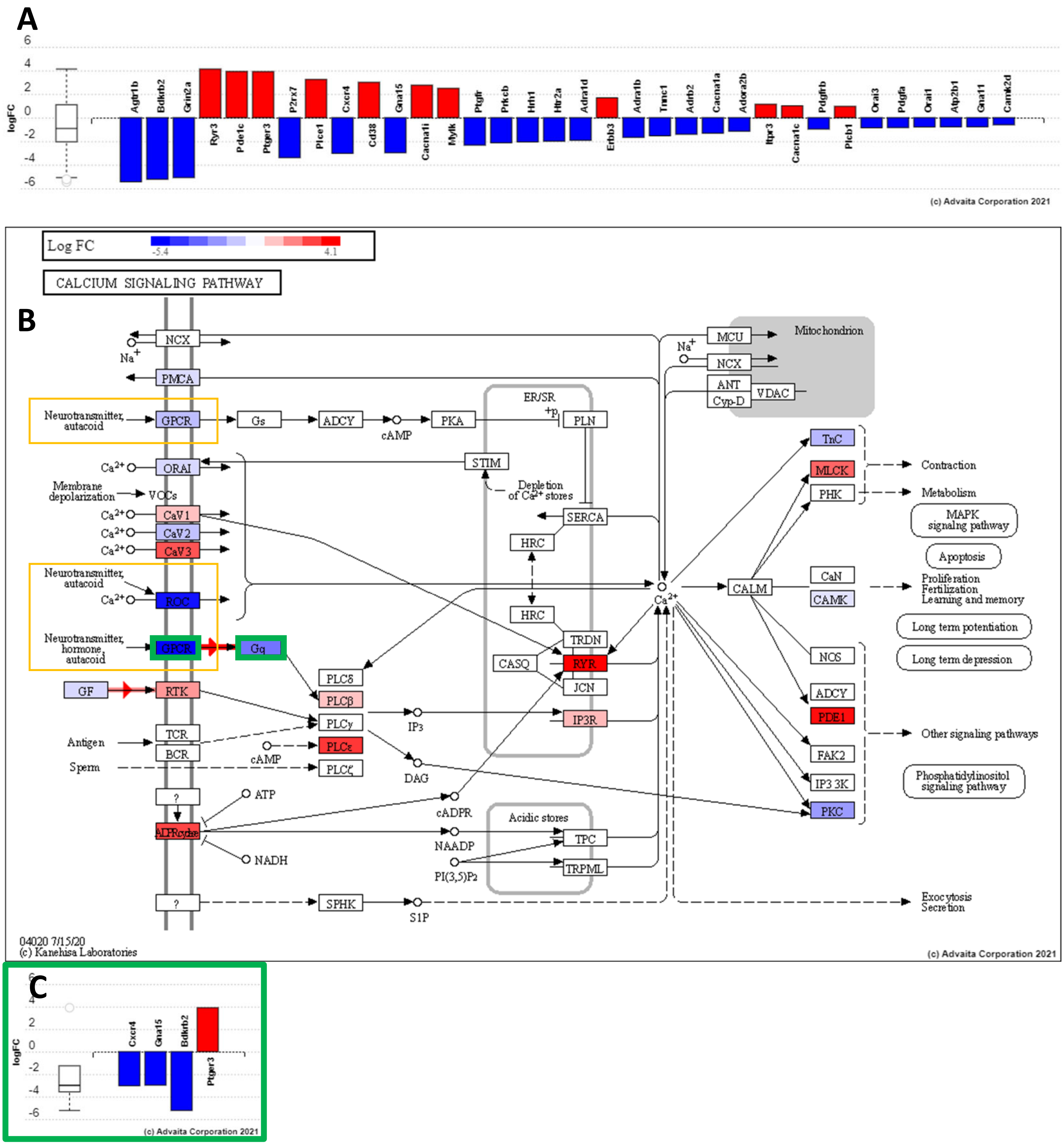

**Figure S10: Neuroactive ligand-receptor interaction. (A)** DEGs in hIGFBP5 fibroblasts that belong to this pathway. **(B)** KEGG pathway showing the logFC of DEGs in (A). Red: upregulated, blue: downregulated, yellow boxes highlight type of ligands, green: hub gene(s). **(C)** Barplot of the logFC of hub gene(s).

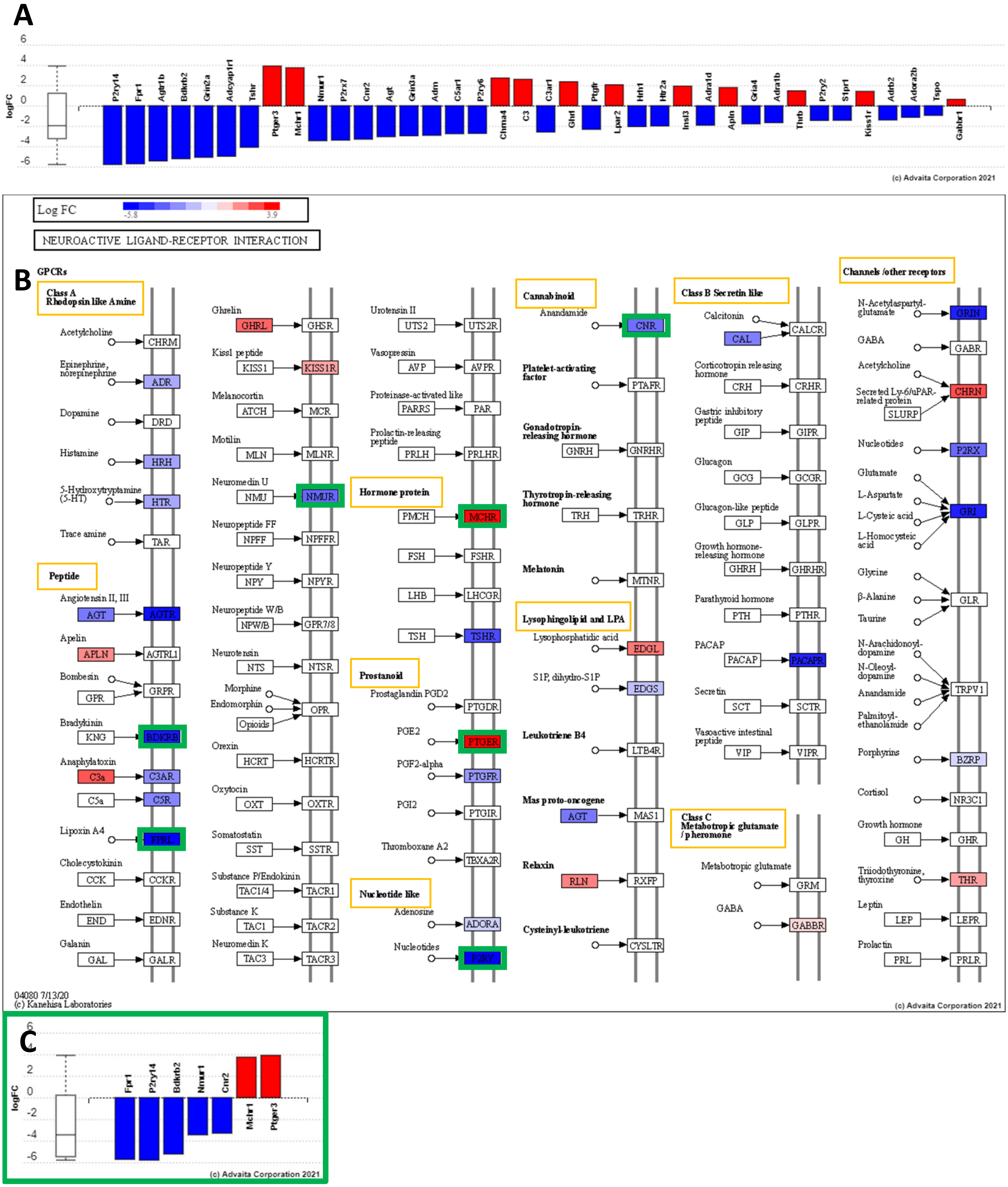

**Figure S11: Impact of hIGFBP5 on the mRNA levels of genes of interest in pFBs.** Expression levels of genes of interest were quantified by qRT-PCR and normalized to female WT for **(A)** *Col27a1*, **(B)** *Pcolc2*, **(C)** *Bdkrb2,* **(D)** *Cav3*, **(E)** *Spp1*, **(F)** *Nmur1* and **(G)** *Il6*. N= 3 per group. The housekeeping gene used to normalize the data was B2m. *P < 0.05. **P < 0.01. ***P < 0.001. ****P < 0.0001. ns: not significant. F: female (in purple), M: male (in green), WT: wild type, HOMO: hIGFBP5 homozygous mice.

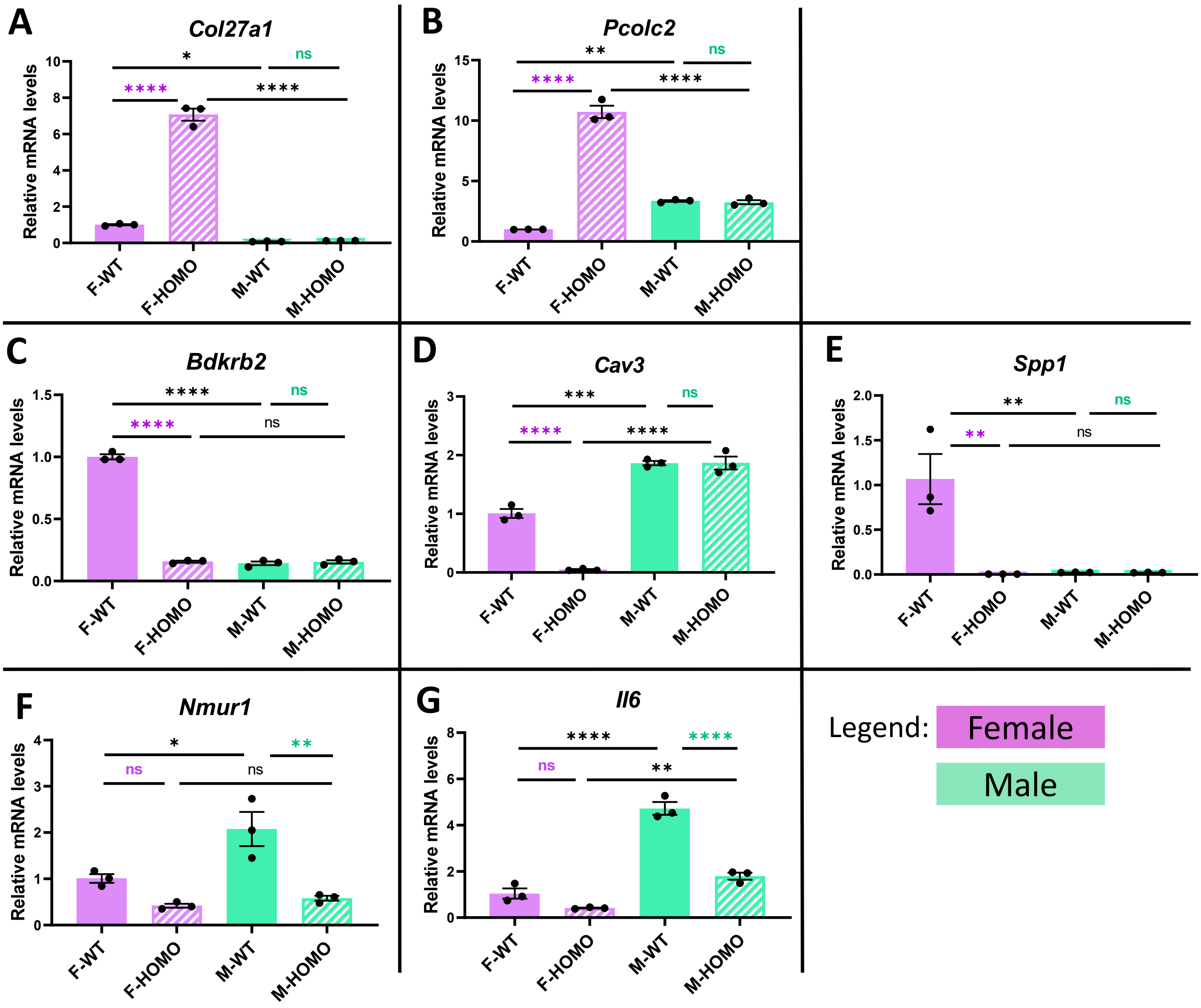

**Figure S12: Effect of hIGFBP5 on Intracellular Protein Abundance.** Cell lysates were analyzed by immunoblotting for selected proteins. Quantification of immunoblots (n=3) for the protein abundance of **(A)** Nmur1, **(B)** Bdkrb2, **(C)** Serpine1, **(D)** Fpr1 and **(E)** Tbx1 was performed, and the results were normalized to female WT. The housekeeping protein used as loading control was β-actin. *P < 0.05. **P < 0.01. ***P < 0.001. ns: not significant. F: female (in purple), M: male (in green), WT: wild type, HOMO: hIGFBP5 homozygous mice. **(F)** Representative immunoblots for each protein shown in panels A-E and β-actin, as a loading control.

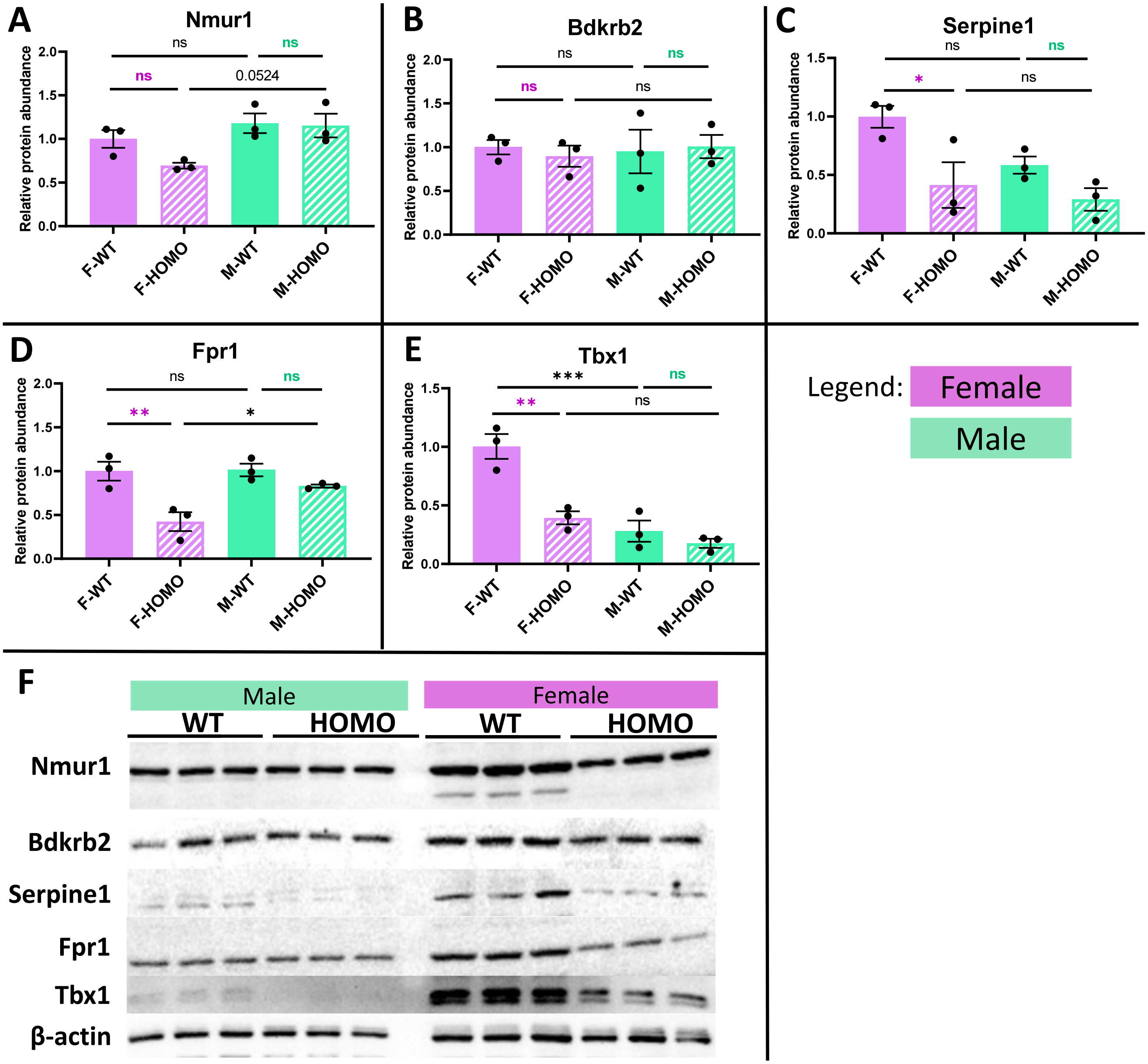

**Figure S13:. Effect of hIGFBP5 on the secretion of Igfbp4, Serpine1 and Pappa2.** Conditioned media of cultured pFBs of hIGFBP5 HOMO and WT mice were analyzed by immunoblotting for selected proteins. Quantification of immunoblots (n=3) for the protein abundance of **(A)** Igfbp4, **(B)** Serpine1 and **(C)** Pappa2 were normalized to female WT. *P < 0.05. **P < 0.01. ***P < 0.001. ****P < 0.0001. ns: not significant. F: female (in purple), M: male (in green), WT: wild type, HOMO: hIGFBP5 homozygous mice. **(D)** Representative immunoblots showing protein abundance of Igfbp4, Serpine1 and Pappa2 in conditioned media of cultured pFBs from hIGFBP5 HOMO and WT mice. Ponceau S staining was used as loading control.

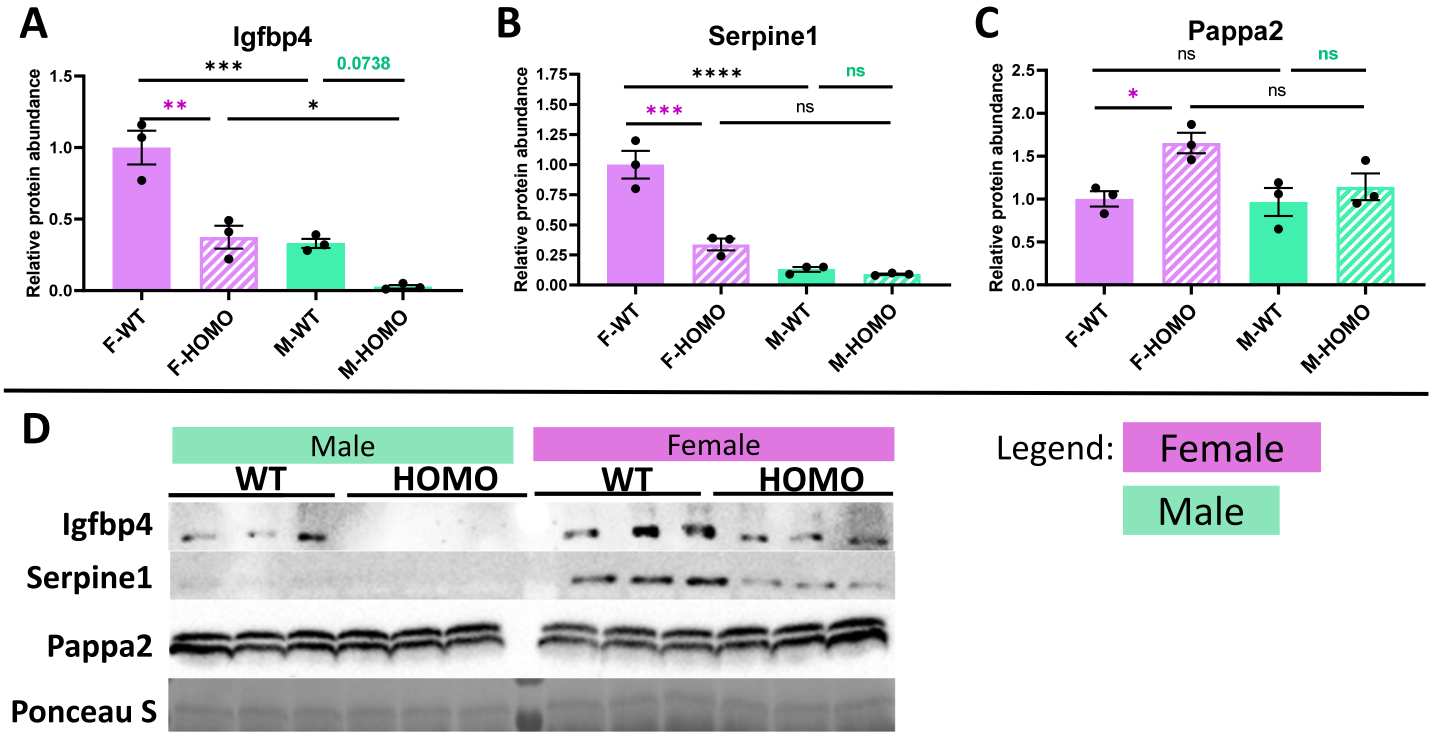

Supplement: Supplementary file 1 [file ijms-22-12609-s001.zip › ijms-1412010-supplementary.pdf]
